# Testing the Generalizability of the Priority Heuristic to Two-Stage Decision Making Tasks: A Comparison to Quantum Cognition and Prospect Theory

**DOI:** 10.1007/s42113-025-00260-w

**Published:** 2025-12-24

**Authors:** Christopher R. Fisher, Othalia Larue, Kevin Schmidt

**Affiliations:** 1 Parallax Advanced Research, Beavercreek, OH 45324 USA; 2 Air Force Research Laboratory, Wright Patterson AFB, OH 45433 USA

**Keywords:** Decision-making, Heuristics, Dynamic-inconsistency, Priority-heuristic, Prospect theory, Quantum cognition

## Abstract

Our goal was to test the ability of the priority heuristic (PH) to generalize to a two-stage decision making task designed to investigate dynamic inconsistency, a phenomenon whereby decision makers deviate from their plans. The PH is a non-compensatory decision strategy in which features are evaluated sequentially in order of importance until one option is determined to be superior. We extended the PH by incorporating reference point dependence into the valuation process and tested it against data from a previously published two-stage decision task (Barkan, R., & Busemeyer, J. R. *Journal of Behavioral Decision Making*, 16(4), 235–255 2003). Although we demonstrated that the extended PH model can produce dynamic inconsistency, further analysis using a true and error model revealed two deficiencies: (1) the model systematically underestimated risk taking preference, and (2) the data violated a critical inequality derived from the model. In a second analysis, we developed a variant which incorporates individual differences, and compared it to two existing models—one based on prospect theory and another based on quantum cognition. Our results show strong evidence for the quantum cognition model over the extended priority heuristic and the model based on prospect theory. Taken together, our results cast doubt on the ability of the PH to generalize to two-stage decision making.

## Introduction

A central question in decision making is how people evaluate and compare options when making a decision. Broadly speaking, theories can be divided into two types: compensatory and non-compensatory. A defining property of compensatory theories is that all features of a given option are integrated into an overall valuation such that a positive feature can offset a negative feature and vice versa. By contrast, non-compensatory theories posit that decision makers only consider a subset of features while ignoring other features regardless of their values. In many cases, the subset of features under consideration is compared to aspiration thresholds representing minimally acceptable values.

Many compensatory theories are based on the ubiquitous notion of expected utility whereby the features of an option are weighted and summed into an overall valuation, and the option with the highest such value is the most likely to be selected. The origins of expected utility can be traced back to Bernoulli during the 18th century (Bernoulli, 1738), with the modern formulation of expected utility theory being axiomatized as a theory of rational choice (Von Neumann & Morgenstern, 1944). In the intervening time, the notion of expected utility spread to cognitive science, where modifications such as loss aversion (Tversky & Kahneman, 1992) and configural weighting (Birnbaum, 2008b, 1974) were incorporated into the basic framework to provide a more accurate description of human decision making, which often fails to satisfy rational axioms (Allais, 1953; Birnbaum, 2008a; Kahneman & Tversky, 1984). As an example of expected utility, consider the choice between a relatively safe gamble $\mathcal {S} = (60,.50; 40,.50)$ representing a 50-50 chance for 60 or 40 dollars, and a relatively risky gamble $\mathcal {R} = (80,.50; 20,.50)$ representing a 50-50 chance for 80 or 20 dollars. When evaluating the expected utility of each option, all information is integrated into a single value: ${{\,\mathrm{\mathbb {E}}\,}}[\mathcal {S}] =.50 \cdot u(60) +.50 \cdot u(40)$, and ${{\,\mathrm{\mathbb {E}}\,}}[\mathcal {R}] =.50 \cdot u(80) +.50 \cdot u(20)$, where utility function *u* transforms objective values in to subjective values.

**Fig. 1:**
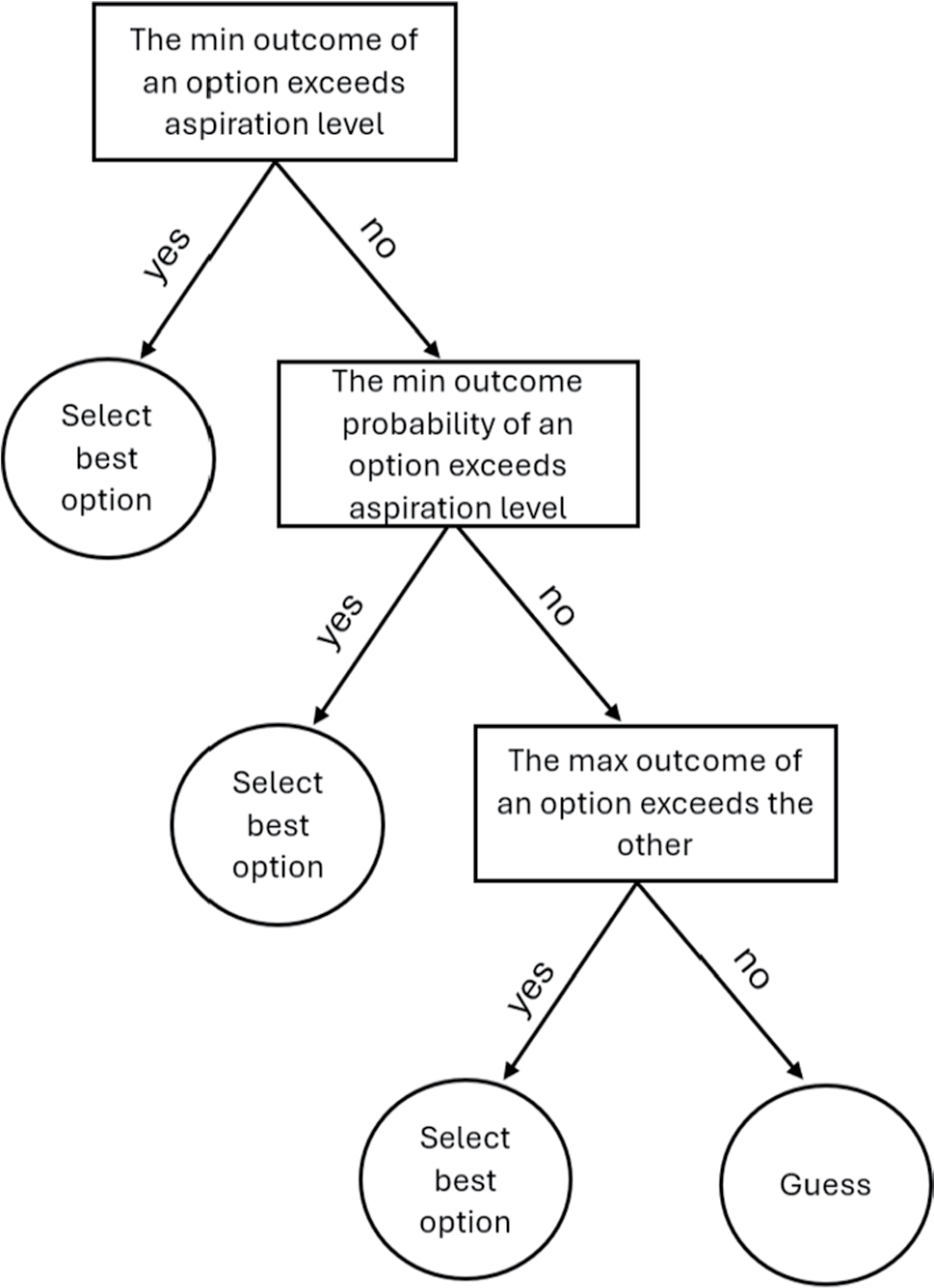
An illustration of the priority heuristic. Starting at the top, options are compared in terms of features ranked in descending order of importance. For a given feature, an option is selected if it has the best value above an aspiration level. Otherwise, the options are compared in terms of the next most important feature

One well-known framework based on non-compensatory decision making is the fast and frugal heuristics approach (Gigerenzer & Todd, 1999). According to the fast and frugal heuristics approach, people draw upon a *toolbox* of heuristics—or simple rules—to quickly extract a small amount of diagnostic information to make a decision. Each heuristic is applicable to a limited set of conditions; hence, the metaphor of a toolbox. Drawing upon bounded rationality, the argument for the fast and frugal approach is that in complex, real world environments it is not feasible to examine and optimally weigh all features to arrive at a decision, nor is it necessary to do so. In this article, we will focus on a fast and frugal heuristic applied to difficult (i.e., ratio of expected values less than or equal to 2) monetary gambles called the priority heuristic PH;, (Brandstätter et al., 2006). According to the PH, people sequentially compare the features between options in descending order of importance, and select an option as soon as one is sufficiently better in terms of a given feature. As illustrated in Fig. 1, these features are ordered as minimum outcomes, minimum outcome probabilities, and maximum outcomes. Returning to the previous example, the PH begins by comparing the minimum outcomes of each gamble (40 vs. 20) to an aspiration threshold equal to $10\%$ of the maximum outcome (80). In this case, the PH selects $\mathcal {S}$ without consideration of the other features because $40 - 20> 80 \cdot .10$.

Brandstätter et al. (2006) presented several lines of evidence in support of the PH’s ability to account for several key decision making phenomena, including the fourfold pattern, the certainty effect, and the Allais Paradox. In a model comparison, the authors found that the PH performed at least as well as other models on several datasets involving binary gambles. Finally, the authors found some evidence for sequential evaluation of features—namely, the median response times increased with the predicted number of features people considered before making a decision.

Subsequent analyses have cast doubt on the PH. For example, when the parameters of alternative models were properly estimated, the alternative models performed similarly and sometimes better than the PH (Birnbaum, 2008a). Moreover, the PH performed poorly compared to alternative models when applied to more diagnostic choice sets, such as gambles with three outcomes instead of two (Birnbaum, 2008a). Along similar lines, Glöckner and Betsch (2008) designed more diagnostic choice sets for testing the PH, and found that prospect theory provided a more accurate account in many cases. Hilbig (2008) tested the following hypothesis derived from the PH: if one feature is sufficient to make a decision, then decision times should not vary with the number of features pointing to the selection of the same option. Contrary to the PH, Hilbig (2008) found that decision times do vary with the number of features pointing to the option predicted by the PH. Furthermore, Birnbaum (2023) found that empirical data violated a critical property of the PH called interactive independence, according to which an effect of one feature difference is independent of other features which are equal in value across alternatives.

In this paper, we apply an alternative strategy to test the PH: we examine its ability to generalize to a more complex two-stage decision making task reported in Barkan and Busemeyer (2003). In this task, subjects often exhibit *dynamic inconsistency* (DI), a phenomenon whereby planned decisions for the second stage are subsequently changed after observing the outcome from the first stage (Barkan & Busemeyer, 2003). Thus, our goal is to investigate whether the PH can generalize to the two-stage decision making task and account for DI. Given that theorists have argued that fast and frugal heuristics are not universally applicable (Gigerenzer & Todd, 1999; Brandstätter et al., 2006), one important question is whether the PH is applicable to the two-stage decision making task. Our position is that the PH is applicable to the two-stage decision making task because (1) it is a simple extension of the single stage decision tasks to which the PH is commonly applied and (2) the decisions are considered difficult according to several alternative measures discussed below.

Another question that arises is why is the two-stage decision making task is an interesting test case for the PH? One reason is because DI violates the normative principle of dynamic consistency (Barkan & Busemeyer, 2003; Johnson & Busemeyer, 2001). Cognitive science has a long history of uncovering and explaining violations of rational models e.g.,, (Tversky and Kahneman, 1992; Birnbaum, 2008a; Costello and Watts, 2014; Roe et al., 2001). Hence, we ask whether the PH can provide a competing, or perhaps superior account of DI? Another reason the two-stage decision task is an interesting test case is because a minor change to a task may create a compelling challenge for a model. In this case, the same choices are presented twice and a planned decision is included. If the PH fails to generalize to a simple extension of the typical monetary gamble paradigm, it would cast more doubt than if it failed to generalized to a drastically different decision making task.

## Overview

The remainder of this paper is structured as follows. We begin by describing the two-stage decision task and DI, which is often observed in the two-stage decision task. Next, we describe the PH as it is applied to simple decision tasks involving a single decision stage. In the next section, we extend the PH to the two-stage decision task using a concept called reference point dependence e.g.,, (Tversky and Shafir, 1992). In so doing, we demonstrate that the extended PH, termed reference point priority heuristic (RPPH), predicts dynamic inconsistency under specific conditions. However, when comparing the RPPH to data from previously published two-stage decision task (Barkan & Busemeyer, 2003), we found that the data are largely at odds with the RPPH. Next, we generalize the RPPH so that its key parameters can vary across individuals and compare it quantitatively with two previously published models—one based on quantum cognition (Busemeyer et al., 2015) and another based on prospect theory (Barkan & Busemeyer, 2003). To preview our main result, Bayesian analysis provides more support for the quantum cognition model compared to the other two models.

## Two-Stage Decision Task

We tested the generalizability of the PH with data from the two-stage decision task reported in Barkan and Busemeyer (2003). A total of 100 subjects completed the experiment and received monetary incentive for their decisions (Barkan & Busemeyer, 2003). Subjects completed one practice trial and 16 test trials (see Table 1). The test trials were presented twice, once in two separate blocks. Additional details can be found in the original paper (Barkan & Busemeyer, 2003).

Each trial consisted of two sequential conditions: a planned decision condition followed immediately by a final decision condition (Barkan & Busemeyer, 2003). The conditions were identical except for whether the decision in the second stage was made in advance (i.e., planned decision condition), or was made after experiencing the outcome from the first stage (i.e., final decision condition).

For ease of exposition, we will describe the final decision condition first, and then explain the change in procedure in the planned condition. In the final condition, there were two decision stages, each featuring the same safe option $\mathcal {S} = (0, 1)$ and risky option $\mathcal {R} = (x_G,.50; x_L,.50)$, where $x_G \ge 0$ is a gain, $x_L \le 0$ is a loss. In the first stage, subjects were required to select $\mathcal {R}$. Next, the computer sampled a random outcome $x_i \in \{ x_G,x_L\}$ from $\mathcal {R}$ and presented it to the subjects. In the second stage, subjects chose between $\mathcal {S}$ and $\mathcal {R}$. Note that $\mathcal {S}$ was the same throughout the experiment. By contrast, $\mathcal {R}$ differed across trials (see Table 1), but was the same across conditions and stages within the same trial. Unless it is unclear from context, we suppress the stage and trial index for simplicity.

As noted above, the planned condition was identical to the final condition, except the decisions were made in advance (Barkan & Busemeyer, 2003). In the planned decision condition, subjects made two planned decisions for the second stage: one contingent upon receiving $x_L$ in the first stage, and the other contingent upon receiving $x_G$ in the first stage. Following Busemeyer et al. (2015), we restrict our analysis to planned and final decisions in which the first stage outcomes match.

## Dynamic Inconsistency

One of the primary goals of the two-stage decision task is to examine whether participants change their planned decisions after observing the outcome from the first stage in the final decision condition. The phenomenon whereby planned decisions differ from final decisions is termed *dynamic inconsistency* DI;, (Busemeyer et al., 2015). Assuming a closed system in which the participant knows all relevant information pertaining to the decision, expected utility theory predicts that subjects make the same decision in both conditions (Johnson & Busemeyer, 2001). The solution for identifying the optimal decision in multi-stage decision making is obtained through a process called backward induction (Johnson & Busemeyer, 2001). Backward induction involves constructing a decision tree spanning the decision stages, where nodes represent decision points and possible outcomes, and edges represent possible pathways. The optimal solution is obtained by pruning inferior options, starting at the last stage, and moving successively up the decision tree until only one sequence of decisions remains. Importantly, backward induction is predicated on the assumption of *dynamic consistency*—the notion that preferences are stable across time. Consequentially, backward induction entails that planned and final decisions must be the same.

## Previous Modeling Efforts

Various models have been proposed to account for DI in multi-stage decision making tasks. Here we will briefly review two successful models which we include in our second model analysis below. The reference point model, which is based on prospect theory, attributes DI to the use of different reference points in the planned and final conditions (Barkan & Busemeyer, 2003; Tversky & Shafir, 1992). According to the reference point model, the hypothetical first stage outcome is not considered in the second stage of the planned condition. However, in the final decision condition, the experienced outcome from the first stage is considered in the second stage, which leads to different predictions. An alternative model based on quantum cognition assumes that DI arises from the way uncertainty in the first stage outcome in the planned condition interferes with a wave function representing the vacillating preferences of the decision maker (Busemeyer et al., 2015). In the planned condition, subjects are in an uncertain state because the outcome has not been observed. However, in the final condition, the uncertainty is resolved because the outcome has been observed. Observing the outcome creates different quantum dynamics, which leads to DI. In a comparison between these models, the data provided more support for the quantum cognition model (Busemeyer et al., 2015).

## Priority Heuristic

To lay the foundation for extending the PH to two-stage decision tasks, we describe the PR in its most basic form as it applies to decisions involving a single stage. Figure 1 illustrates the decision logic of the PH as a tree diagram. In the diagram, the features are ordered from top to bottom in descending order of importance: (1) minimum outcome, (2) minimum outcome probability, and (3) maximum outcome. As shown in the diagram, options are compared one feature at a time until the comparative advantage of one option exceeds an aspiration level on a given feature (Brandstätter et al., 2006). Unlike models based on expected utility theory, the PH is a non-compensatory strategy because a decision can be made without considering all features.

### Features

As a simple example, consider the gamble with only positive values: $\mathcal {G} = (5,.90; 10,.10)$. In this example, the minimum outcome is 5, the minimum outcome probability is.90, and the maximum outcome is 10. Outside of this simple example, the definition of minimum outcome and maximum outcome differs somewhat from the conventional, mathematical definition of minimum and maximum (Brandstätter et al., 2006; Rieskamp, 2008). General definitions that apply to gambles with any outcome, including mixed gains and losses, have not been described formally. In what follows, we formalize our interpretation of minimum outcome and maximum outcome based on verbal descriptions in prior research (Brandstätter et al., 2006; Rieskamp, 2008).

For outcome vector $\textbf{x}$ of a given option, the minimum outcome is defined formally with the function:

1 $$\begin{aligned} h_\text {min}(\textbf{x}) = {\left\{ \begin{array}{ll} x_1 \text { if } x_2 \text { is not defined} \\ \max (x_1, x_2) \text { if } x_1 \le 0, x_2 \le 0\\ \min (x_1, x_2) \text { otherwise} \end{array}\right. }. \end{aligned}$$

What this means is that the smallest loss is the minimum outcome when both values are non-positive, but the minimum value is the minimum outcome when both values are positive or mixed. In cases in which a gamble has only one outcome, e.g., $\mathcal {S} = (x_1, 1)$, $x_1$ is the minimum outcome. For the vector of outcome probabilities $\textbf{p}$ of a given option, the minimum outcome probability is the probability associated with the minimum outcome:

2 $$\begin{aligned} h_\text {pr}(\textbf{p}) = {\left\{ \begin{array}{ll} p_1 \text { if } x_2 \text { is not defined} \\ p_1 \text { if } x_1 = h_\text {min}(\textbf{x}) \\ p_2 \text { otherwise} \end{array}\right. }. \end{aligned}$$

The maximum outcome of a gamble is defined according to the function:

3 $$\begin{aligned} h_\text {max}(\textbf{x}) = {\left\{ \begin{array}{ll} x_1 \text { if } x_2 \text { is not defined} \\ \min (x_1, x_2) \text { if } x_1 \le 0, x_2 \le 0 \\ \max (x_1, x_2) \text { otherwise} \end{array}\right. }. \end{aligned}$$

In this case, the largest loss is used for non-positive values, but the maximum value is used for positive values or mixed values. Similarly, $x_1$ is the maximum outcome when a gamble only has a single outcome.

### Evaluation Rules

The aspiration level is the minimum difference between the feature values of two options required to make a decision. If the aspiration level is met or exceeded, the option with the superior feature value is selected (Brandstätter et al., 2006). Otherwise, the options must be compared with respect to the next most important feature. The aspiration level for the minimum outcome is defined relative to other outcomes, rather than an absolute value. In particular, the aspiration level is defined as $\delta =.10$ of the outcome with largest magnitude across all options. The outcome with the largest magnitude is defined as:

4 $$\begin{aligned} x_{\text {max}} = \max (\{ |x_{i,j} |\}_{i \in \mathcal {I}, j \in \mathcal {J}}), \end{aligned}$$

where $\mathcal {I}$, and $\mathcal {J}$ are index sets for outcomes and options, respectively. The quantity $x_{\text {max}}$ is often rounded to the nearest prominent number, which is defined as a power of 10, or half or twice a power of ten. Following Rieskamp (2008), we omit this step for simplicity without significantly altering predictions. For the probability of the minimum outcome, the aspiration level is $10\%$ of the maximum of the probability scale, which is $\delta \cdot 1 =.10$.

The decision rule for comparing minimum outcomes defined as:

5 $$\begin{aligned} {\left\{ \begin{array}{ll} \text {choose safe if } (h_\text {min}(\textbf{x}_S) - h_\text {min}(\textbf{x}_R) ) \ge \delta \cdot x_{\text {max}} \\ \text {choose risky if } (h_\text {min}(\textbf{x}_R) - h_\text {min}(\textbf{x}_S) ) \ge \delta \cdot x_{\text {max}} \\ \text {check next feature otherwise} \end{array}\right. }, \end{aligned}$$

where *S* and *R* index the safe and risky options, respectively. The decision rule for comparing the minimum outcome probabilities defined as:

6 $$\begin{aligned} {\left\{ \begin{array}{ll} \text {choose safe if } (h_{\text {pr}}(\textbf{p}_R) - h_{\text {pr}}(\textbf{p}_S)) \ge \delta \\ \text {choose risky if } (h_{\text {pr}}(\textbf{p}_S) - h_{\text {pr}}(\textbf{p}_R)) \ge \delta \\ \text {check next feature otherwise} \end{array}\right. }. \end{aligned}$$

Note that the option with the smallest minimum outcome probability is superior. Finally, the decision rule for comparing maximum outcomes is defined as:

7 $$\begin{aligned} {\left\{ \begin{array}{ll} \text {choose safe if } h_\text {max}(\textbf{x}_S)> h_\text {max}(\textbf{x}_R) \\ \text {choose risky if } h_\text {max}(\textbf{x}_R)> h_\text {max}(\textbf{x}_S) \\ \text {guess otherwise} \end{array}\right. }. \end{aligned}$$

It should be noted that in its current formulation the PH cannot account for dynamic inconsistency in participants’ decisions. This limitation can be attributed to the fact that the decision process of the PH does not vary with experimental condition. Next, we turn to a proposed solution for producing dynamic inconsistency with the PH, which entails a modification to the valuation process while maintaining its core decision logic.

## Reference Point Priority Heuristic

In this section, we explain how the PH can be extended to account for dynamic inconsistency in the two-stage decision making task using *reference point dependent valuation*. This concept formed the basis for an extension of prospect theory called the reference point model, which was used to account for dynamic inconsistency (Barkan & Busemeyer, 2003) and similar effects (Tversky & Shafir, 1992). As the name implies, reference point dependent valuation allows one to compare options using a reference point that depends on experimental condition.

Reference point dependent valuation works as follows: In the planned decision condition, the risky option $\mathcal {R}$ and the safe option $\mathcal {S}$ are evaluated without considering the hypothetical outcome $x_i$ in the first stage. Consequentially, the valuations of the outcomes are $\{x_G, x_L\}$ for $\mathcal {R}$, and $\{0\}$ for $\mathcal {S}$. By contrast, in the final decision condition, the realized outcome $x_i$ from the first stage is incorporated into the valuation of the options, producing $\{x_i + x_G, x_i + x_L\}$ for the risky option $\mathcal {R}$, and $\{x_i\}$ for the safe option $\mathcal {S}$. One rationale for omitting the hypothetical outcome in the planned decision condition is that it may have lower salience or emotional impact compared to experienced outcomes, causing the decision maker to completely discount the hypothetical outcome. Henceforth, we will refer to this extension as the reference-point priority heuristic (RPPH).

**Fig. 2:**
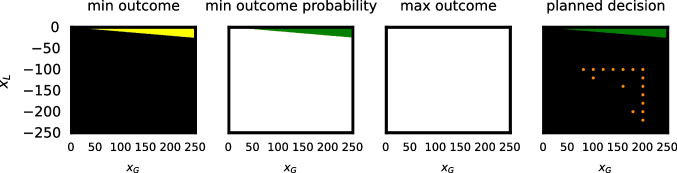
Predictions of the reference point priority heuristic (RPPH) for the planned decision condition. Notes: Actions are color coded as black: select safe option, green: select risky option, yellow: check next feature, and white: decision made in a previous stage. Orange dots represent gamble outcomes used in Barkan and Busemeyer (2003). The first three plots represent the *internal* processes of the RPPHs. The last plot is the predicted planned decision collapsed across stages. $p_{1,2} =.50$ for first outcome second stage

**Fig. 3:**
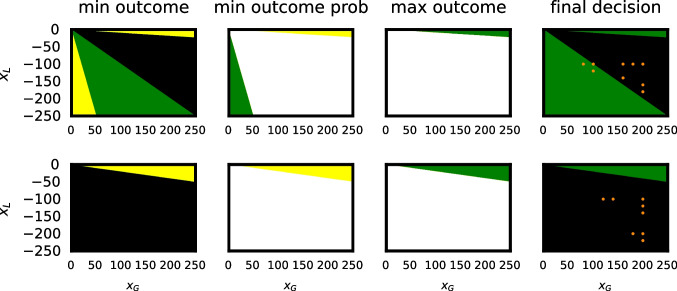
Predictions of the extended reference point priority heuristic for the final decision condition. Top row is conditioned on a loss ($x_L$) in stage 1. Bottom row is conditioned on a gain ($x_G$) in stage 1. See notes in Fig. 2 for details

One consideration we must make is whether the PH is applicable to the gambles in Table 1. As Brandstätter et al. (2006) noted, the PH is only applicable to difficult choices with similar expected values. For expected values with the same sign, this is commonly defined as $\frac{{{\,\mathrm{\mathbb {E}}\,}}(\mathcal {R})}{{{\,\mathrm{\mathbb {E}}\,}}(\mathcal {S})} \le 2$. This measure of decision difficulty presents several challenges for the gambles used in Table 1 because it is undefined for planned decisions (e.g., dividing by ${{\,\mathrm{\mathbb {E}}\,}}(\mathcal {S}) = 0$) and the expected values have mixed signs in some cases. Here, we consider two alternative measures. First, we consider stochastic dominance. In the choice sets used in (Barkan & Busemeyer, 2003), neither option stochastically dominates the other, suggesting the decisions are not easy. Second, we consider the following signal-to-noise measure between the two options:

8 $$\begin{aligned} \bigg |\frac{{{\,\mathrm{\mathbb {E}}\,}}(\mathcal {R}) - {{\,\mathrm{\mathbb {E}}\,}}(\mathcal {S})}{\sqrt{\textrm{Var}(\mathcal {R}) + \textrm{Var}(\mathcal {S})}} \bigg | . \end{aligned}$$

According to this measure, smaller values in Eq. 8 indicate more decision difficulty because it is challenging to discern which option yields a greater payout on average. One benefit of the signal-to-noise ratio is that it is defined when one option has an expected value of zero and if the expected values have different signs. As shown in Table 1, the gambles used in Barkan and Busemeyer (2003) are considered difficult according to this alternative measure.

### Predictions

In the following sections, we will use the contour plots in Figs. 2 and 3 to analyze the internal decision process of the RPPH and its predictions for DI. Each contour plot represents the predictions of the RPPH within a $x_G \times x_L$ stimulus space encompassing the gambles used in Barkan and Busemeyer (2003). The contour plots can be obtained by substituting the gamble features into Eqs. 1, 3, and 4, and substituting the resulting values into the decision rules defined in Eqs. 5, 6, and 7.

The internal decision process can be traced by reading the first three contour plots from left to right: minimum outcome, minimum outcome probability, and maximum outcome. The contour plots in the fourth column represent the predicted decision collapsed across the three feature evaluation stages.

#### Internal Decision Process

Inspection of the first three contour plots reveals that a decision is made based on the minimum outcome for most of the stimulus space. The decisions occurring in the minimum outcome stage do, however, differ by condition: in both the planned decision condition (see Fig. 2) and the final decision condition with $x_G$ as the first stage outcome (see Fig. 3), the RPPH largely predicts selection of the safe option. By contrast, in the final condition with $x_L$ as the first stage outcome, the space is split largely between the selection of the safe option in the region defined by $|x_L| < x_G$ and the selection of the risky option in the region defined by $|x_L| \ge x_G$. These differences across conditions are due to reference point dependence.

#### Dynamic Inconsistency Predictions

The predictions for dynamic inconsistency can be gleaned by comparing fourth contour plot in the planned decision condition (see Fig. 2) to the fourth contour plots of the final decision conditions (see Fig. 3). One key insight is that the RPPH can produce dynamic inconsistency in some regions of the prediction space. When the first stage outcome is $x_G$, the RPPH predicts dynamic inconsistency for a narrow region located at the top right of the prediction space. By contrast, when the first stage outcome is $x_L$, the RPPH predicts dynamic inconsistency in a much larger region, defined by $|x_L| \ge x_G$. For the gambles used in Barkan and Busemeyer (2003), the RPPH does not predict dynamic inconsistency for outcome $x_G$. Instead, it predicts that people will select the safe option for both planned and final decisions. However, for three of nine gambles, the RPPH does predict dynamic inconsistency when the first stage outcome is $x_L$, such that people shift from the safe option in the planned condition to the risky option in the final decision. In the remaining gambles, the RPPH predicts the selection of the safe option for the planned and final decision.

## Analysis Plan

In this section, we outline our model analysis plan, which is organized into two separate analyses—one for the RPPH and a another for a generalization of the RPPH called the stochastic reference point priority heuristic (*SRPPH*). Much like the RPPH, the SRRPH incorporates reference point dependence, but building upon the generalization of the PH by Rieskamp (2008), it also incorporates individual differences in aspiration levels and stochasticity in decision making. In short, we organize the paper into two separate analysis because RPPH and *SRPPH* use different analyses performed at different levels of the data.

Our first analysis examines the predictions of the RPPH, which is of theoretical interest because it closest to the original instantiation of the PH i.e.,, (Brandstätter et al., 2006). The RPPH simply incorporates reference point dependence to the valuation process to produce DI. Much like the PH, the RPPH makes deterministic predictions and has no free parameters. One challenge in evaluating the RPPH is that it makes deterministic predictions but prior research shows that decision making is inherently stochastic (Rieskamp, 2008). Without an error model, the threshold for falsifying the RPPH with one incompatible observation would be unrealistically low. To overcome this difficulty, we evaluate the RPPH with a true and error model TEM;, (Birnbaum, 2013) to provide a flexible error theory within minimal assumptions. An important restriction is that the TEM requires pooling across subjects and performing a separate analysis for each trial.

Our second analysis examines the predictions of the *SRPPH* at the individual level. As noted above, the *SRPPH* allows individual variability in aspiration levels and stochasticity in decision making. In contrast to the TEM, one potential downside, is that the Thurstonian approach commits to a more restrictive error model based on Gaussian distributions. In the next section, we provide an brief overview of the Bayesian framework in which both analyses are conducted.

### Bayesian Framework

Our model analyses below were performed within a Bayesian statistical framework. The benefits of using Bayesian methods have been detailed in many sources e.g.,, (Wagenmakers et al., 2018; Kruschke, 2011; Vanpaemel, 2010; Wagenmakers, 2007; Jaynes, 2003). To summarize, some of the benefits include, the ability to characterize prior uncertainty in models and/or model parameters as probability distributions, update these sources of uncertainty in light of new data, and the ability to account for multiple sources of model complexity during model comparison, including parameters and functional form. In the following sections, we will provide a brief overview of the Bayesian framework to lay the groundwork for our analyses.

#### Bayesian Parameter Estimation

Bayesian parameter estimation allows one to quantify initial uncertainty in parameter values with a prior distribution over the parameters and update the prior distribution based on observed data, resulting in a posterior probability distribution of the parameters. We begin by defining $\mathcal {M}$ as a model with parameter vector $\boldsymbol{\theta }$. The prior density for the parameters is given by $p(\boldsymbol{\theta } \mid \mathcal {M})$, and *f* is the probability density function (or a probability mass function in the case of discrete data) for $\mathcal {M}$. The posterior density of $\boldsymbol{\theta }$ given observed data vector $\textbf{Y}$ is defined as:

9 $$\begin{aligned} p(\boldsymbol{\theta } \mid \textbf{Y}, \mathcal {M}) = \frac{f(\textbf{Y} \mid \boldsymbol{\theta }, \mathcal {M}) p(\boldsymbol{\theta } \mid \mathcal {M})}{\int _{\boldsymbol{\theta }\in \boldsymbol{\Theta }} f(\textbf{Y} \mid \boldsymbol{\theta }, \mathcal {M}) p(\boldsymbol{\theta } \mid \mathcal {M}) \textbf{d} \boldsymbol{\theta }}, \end{aligned}$$

where $\boldsymbol{\Theta }$ is the parameter space for $\mathcal {M}$.

#### Bayesian Model Comparison

As a global index of model fit, we compared the models using Bayes factors. In Bayesian model comparison, the Bayes factor for model $\mathcal {M}_i$ relative to $\mathcal {M}_j$ is related to posterior model odds as follows:

10 $$\begin{aligned} \frac{p(\mathcal {M}_i \mid \textbf{Y})}{p(\mathcal {M}_j \mid \textbf{Y})} = \frac{p(\mathcal {M}_i)}{p(\mathcal {M}_j)} \textrm{BF}_{i,j}, \end{aligned}$$

where the first term on the right hand side is the prior model odds, $\textrm{BF}_{i,j}$ is the Bayes factor comparing model $\mathcal {M}_i$ to model $\mathcal {M}_j$. Inspection of Eq. 10 reveals that the Bayes factor can be interpreted as the factor by which prior odds are adjusted in light of the data. One advantage of Bayes factors is that they can be used even when there is disagreement over the prior model odds, provided there is agreement over model specification. For example, $\textrm{BF}_{i,j} = 5$ means the data are five times more likely under $\mathcal {M}_i$ than $\mathcal {M}_j$, and the prior odds—whatever they might be—must be adjusted by a factor of 5. The following equation shows that the Bayes factor can be expressed in terms of marginal likelihoods:

11 $$\begin{aligned} \textrm{BF}_{i,j} = \frac{f(\textbf{Y} \mid \mathcal {M}_i)}{f(\textbf{Y} \mid \mathcal {M}_j)}, \end{aligned}$$

where the marginal likelihood of $\mathcal {M}$ is given by:

12 $$\begin{aligned} f(\textbf{Y} \mid \mathcal {M}) = \int _{\boldsymbol{\theta }\in \boldsymbol{\Theta }} f(\textbf{Y} \mid \boldsymbol{\theta }, \mathcal {M}) p(\boldsymbol{\theta } \mid \mathcal {M}) \textbf{d} \boldsymbol{\theta }, \end{aligned}$$

which appears as the normalizing constant in the denominator of Eq. 9. At a conceptual level, the marginal likelihood assesses the ability of a model to predict the data using all points in its parameter space weighted by their prior probabilities. Another benefit of the Bayes factor, which can be attributed to the use of the marginal likelihood, is its ability to penalize flexible models by taking into consideration functional form, dimensionality, and flexibility attributed to the prior over $\boldsymbol{\Theta }$ (Myung & Pitt, 1997). Thus, the Bayes factor balances model fit with model flexibility.

#### Posterior Predictive Distributions

A posterior predictive distribution allows one to predict future data $\textbf{Y}^\prime$ after updating parameters $\boldsymbol{\theta }$ on the basis of observed data $\textbf{Y}$. A posterior predictive distribution for $\mathcal {M}$ is formed by evaluating the likelihood of the data weighted by the posterior density of the parameters, and integrating over the allowable parameter space:

13 $$\begin{aligned} f(\textbf{Y}^\prime \mid \textbf{Y}, \mathcal {M}) = {\int _{\boldsymbol{\theta }\in \boldsymbol{\Theta }} f(\textbf{Y}^\prime \mid \boldsymbol{\theta }, \mathcal {M}) p(\boldsymbol{\theta } \mid \textbf{Y}, \mathcal {M}) \textbf{d} \boldsymbol{\theta }}. \end{aligned}$$

A model has high descriptive adequacy if it reproduces key patterns in the data such that the data fall within high density regions of the posterior predictive distributions. Data that fall outside of high density regions of the posterior predictive distributions reveal deficiencies of the model. In practice, it is beneficial to supplement a Bayes factor by comparing the data to the posterior predictive distribution, as it helps distinguish between a model that describes the data well from one that is merely the least bad model under consideration.

## Analysis 1

The first analysis examines the RPPH with a true and error model (TEM) analysis. Before analysing the RPPH, we introduce the TEM and show how it can be extended to account for potential response dependencies in the two-stage decision task examined by Barkan and Busemeyer (2003).

### True and Error Model Analysis

One challenge with evaluating a deterministic model, such as the RPPH, is the lack of error theory. Given that decision making is subject to numerous sources of error (Rieskamp, 2008), such as lapses of attention, misreading a description, and selecting the incorrect response button, observed responses cannot be interpreted as veridical indicators of a person’s true preference. As an example, suppose a person chooses $\mathcal {R}$ from the choice set $\{\mathcal {R}, \mathcal {S}\}$. There are two mutually exclusive cases: (1) the choice was consistent with his or her true preference for $\mathcal {R}$, or (2) he or she truly preferred $\mathcal {S}$, but selected $\mathcal {R}$ through error. As such, we need a method to disentangle true preferences from errors.

To solve this problem, we evaluated the RPPH with a true and error model (TEM), according to which observed choices reflect a mixture of true preferences and errors (Birnbaum, 2013; Birnbaum, 2023). One important feature of TEMs is that they make minimal assumptions about the error distribution. TEMs, assume that errors are independent and preferences do not change over short periods of time (e.g., during an experimental session), but otherwise make no assumptions regarding the nature or source of errors.^1^ An experiment must satisfy two additional requirements to be analyzed with a TEM: (1) participants must select an option from two choice sets, and (2) participants must select an option from each choice set at least twice. The two-stage decision task described in Barkan and Busemeyer (2003) satisfies both requirements. Participants select an option from choice set $\mathcal {C}_p = \{\mathcal {R}, \mathcal {S}\}$ in the planned condition and choice set $\mathcal {C}_f = \{\mathcal {R}, \mathcal {S}\}$ in the final condition, and these decisions are made twice, separated by other decision trials. However, on each replicate, planned and final decisions are made consecutively within the same trial. As such, a person might simply repeat the planned decision due to a desire to be consistent, leading to an additional source of response dependence. After presenting the TEM in more detail, we will augment it to account for dependence between the planned and final choices within a replication of the same trial.

### Standard TEM

In a TEM, the state space for a decision maker’s true preferences is $\mathcal {P} = \{S_{p}S_{f},S_{p}R_{f},R_{p}S_{f},R_{p}R_{f}\}$, where *S* corresponds to preference for the safe option, *R* corresponds to preference for the risky option, and *p* and *f* correspond to the planned and final decision conditions, respectively. The joint probability distribution over true preference states is given by parameters $p_{\mathrm {R_{p}}{R_{f}}}, p_{\mathrm {R_{p}}{S_{f}}}, p_{\mathrm {S_{p}}{R_{f}}}, p_{\mathrm {S_{p}}{S_{f}}} \ge 0$, subject to the constraint $\sum _{j \in \mathcal {P}} p_j = 1$. The TEM includes four parameters representing error probabilities: $0 \le \epsilon _{\textrm{S}_p},\epsilon _{\textrm{S}_f},\epsilon _{\textrm{R}_p},\epsilon _{\textrm{R}_f} \le .50$, where the first index (R or S) is the erroneously selected option and the second index (p or f) corresponds to the condition. As an example, $\epsilon _{\textrm{R}_p}$ is the probability of erroneously selecting gamble $\mathcal {R}$ in the planned condition while having a true preference for gamble $\mathcal {S}$.

By design, the error probability parameters abstract away the details of error generation—it could be due to a mistake in response execution, an error in comparing or combining features, momentary oscillations in preference, or something else entirely. Unlike some models in which noise is a primary explanatory mechanism e.g.,, (Costello and Watts, 2014), in the TEM noise introduces random variability into the predicted responses and is treated as a nuisance factor. The primary advantage of using the TEM is that it creates a more powerful and direct test of the RPPH by disentangling errors from the central predictions of the RPPH.

**Table 1:** A comparison of the predictions for the RPPH and data for each trial from Barkan and Busemeyer (2003)

id	$x_G$	$x_L$	Outcome	RPPH	STN	$\log _{10}(\text {BF})$
1	200	−200	$x_G$	$S_pS_f$	0.00	−3
2	120	−100	$x_G$	$S_pS_f$	0.09	−11
3	180	−200	$x_G$	$S_pS_f$	0.05	−3
4	200	−120	$x_G$	$S_pS_f$	0.25	−14
5	200	−220	$x_G$	$S_pS_f$	0.05	−6
6	200	−100	$x_G$	$S_pS_f$	0.33	−19
7	140	−100	$x_G$	$S_pS_f$	0.17	−9
8	200	−140	$x_G$	$S_pS_f$	0.18	−7
9	200	−100	$x_L$	$S_pS_f$	0.33	NA
10	160	−140	$x_L$	$S_pS_f$	0.07	−8
11	180	−100	$x_L$	$S_pS_f$	0.29	−19
12	100	−120	$x_L$	$S_pR_f$	0.09	−2
13	200	−160	$x_L$	$S_pS_f$	0.11	−14
14	160	−100	$x_L$	$S_pS_f$	0.23	−14
15	200	−180	$x_L$	$S_pS_f$	0.05	−12
16	80	−100	$x_L$	$S_pR_f$	0.11	−4
17	100	−100	$x_L$	$S_pR_f$	0.00	−7

Columns $x_G$ and $x_L$ correspond to possible gains and losses of the risky option for each trial. Outcome refers to the experienced outcome from stage 1. The column RPPH lists the preference states for RPPH for each trial. *STN *absolute value of signal to noise as a measure of decision difficulty, which is the same in the planned and final decision conditions (see Eq. 8). $log_\mathit{10}\mathit(BF\mathit)$ log Bayes factors comparing RPPH relative to true and error model with a small dynamic inconsistency effect. $log_\mathit{10}(BF)$ NA for practice trial 9 indicates TEM analysis is not applicable because it lacks the required two repetitions

Given two sets of binary choice sets that are repeated twice, there are $2^4 = 16$ joint choice patterns: $\{(R_{p},R_{f}, R_{p},R_{f}), \dots , (S_{p},S_{f}, S_{p},S_{f})\}$. Consequentially, the TEM consists of 16 equations, one defining the probability for each response pattern. As an example, consider the choice pattern $(R_{p},R_{f}, R_{p},R_{f})$. There are four ways this pattern could arise: a person is in preference state $R_pR_f$, $R_pS_f$, $S_pR_f$, or $S_pS_f$, each occurring with its own probability represented by parameters $p_{\mathrm {R_{p}}{R_{f}}}, p_{\mathrm {R_{p}}{S_{f}}}, p_{\mathrm {S_{p}}{R_{f}}}, p_{\mathrm {S_{p}}{S_{f}}}$. The probability of the response pattern is found by taking the product of each preference state and its corresponding response pattern, and summing these quantities across all four preference patterns:

14 $$\begin{aligned} \begin{aligned} \Pr ({R_{p}},{R_{f}},{R_{p}},{R_{f}}) =&\\ &p_{\mathrm {R_{p}}{R_f}} \cdot \left[ (1 - \epsilon _{\textrm{S}_p})\ \cdot\ (1 - \epsilon _{\textrm{S}_f}) \right] ^2+\\&p_{\mathrm {R_{p}}{S_{f}}} \cdot \left[ (1 - \epsilon _{\textrm{S}_p})\ \cdot\ \epsilon _{\textrm{R}_f} \right] ^2+\\&p_{\mathrm {S_{p}}R_{f}} \cdot \left[ \epsilon _{\textrm{R}_p}\ \cdot\ (1 - \epsilon _{\textrm{S}_f}) \right] ^2+ \\&p_{\mathrm {S_{p}}S_{f}} \cdot \left[ \epsilon _{\textrm{R}_p}\ \cdot\ \epsilon _{\textrm{R}_f}\right] ^2 . \end{aligned} \end{aligned}$$

The same logic applies to the remaining 15 equations.

### TEM with Response Dependencies

We extended the TEM to account for possible response dependencies between the planned and final decisions within a repetition of the same trial, forming what we term the True and Error Dependent Model (TEDM). Following the logic Busemeyer et al. (2015) used for other models, we modeled repeated choices (e.g., ($R_p$, $R_f$)) as a mixture of two processes: (1) simply repeating the planned response as the final response with probability $p_{\text {rep}}$, and (2) selecting the same response made in the planned condition through the decision process of the RPPH with probability $(1 - p_{\text {rep}})$. When $p_{\text {rep}} = 0$, the TEDM reduces to the TEM. The full set of equations and a more detailed explanation can be found in Appendix A. Moving forward, we will denote the TEDM for the RPPH as $\mathcal {M}_{\text {RPPH}}$.

#### Critical Inequality

An important part of model evaluation is testing critical properties of a model, such as an equality or relationship that must hold for all permissible parameter values e.g.,, (Birnbaum, 2011). In service of this goal, we briefly describe a critical inequality of the $\mathcal {M}_{\text {RPPH}}$ pertaining to the selection of all risky options and all safe options. According to the critical inequality, when $p_{S_pS_f} = 1$, as is the case for most gambles in Table 1, the following relationship must hold:

15 $$\begin{aligned} \Pr (R_p,R_f,R_p,R_f) \le \Pr (S_p,S_f,S_p,S_f). \end{aligned}$$

A violation of this critical inequality would be considered strong evidence against the $\mathcal {M}_{\text {RPPH}}$ because the critical inequality holds for all allowable parameter values of $\mathcal {M}_{\text {RPPH}}$, including those controlling error probabilities and response dependencies. Appendix B provides a proof demonstrating that the $\mathcal {M}_{\text {RPPH}}$ implies the inequality in Expression 15.

### Connecting RPPH to TEDM

In this section, we connect the RPPH to TEDM to form a testable model we denote as $\mathcal {M}_{\textrm{RPPH}}$. The predictions of the RPPH are specified in the preference state parameters of the TEDM: $\textbf{p} = \left[ p_{\mathrm {R_{p}R_{f}}}, p_{\mathrm {R_{p}S_{f}}}, p_{\mathrm {S_{p}R_{f}}}, p_{\mathrm {S_{p}S_f}} \right]^{\boldsymbol{\intercal}}$. The values for preference state parameters are obtained by applying Eqs. 1-7 to the gamble features in Table 1. Given that the RPPH makes deterministic predictions, the preference state corresponding to the prediction has a value of 1 and the other preference states have a value of 0. The predictions are listed in Table 1 and visualized in Figs. 2 and 3. For example, in row 1 of Table 1, $p_{S_{p}S_{f}} = 1$.

The prior distributions for $\mathcal {M}_{\textrm{RPPH}}$ are specified as follows:

$$\begin{aligned} {\begin{matrix} & p_k = 1, k \in \mathcal {P} \\ & p_i = 0, \forall i \ne k,\text {rep} \\ & p_{\text {rep}} \sim \textrm{uniform}(0, 1) \\ & \boldsymbol{\epsilon } {\mathop {\sim }\limits ^{iid}} \textrm{uniform}(0,.50),\\ \end{matrix}} \end{aligned}$$

where $p_k$ is the preference state for the predicted choice pattern of the RPPH, $p_i$ denotes the preference state of a non-predicted choice pattern, and $\boldsymbol{\epsilon } = \left[ \epsilon _{\textrm{S}_p},\epsilon _{\textrm{S}_f},\epsilon _{\textrm{R}_p},\epsilon _{\textrm{R}_f} \right] ^\intercal$ is the vector of error probability parameters.

We compared the $\mathcal {M}_{\textrm{RPPH}}$ to an alternative model that predicts a small, symmetric DI, denoted $\mathcal {M}_{\textrm{DI}}$. These models differ in their assumptions about preference state probabilities, but make the same assumptions about response dependencies and error probabilities. The prior distribution for the $\mathcal {M}_{\textrm{DI}}$ is given by:

$$\begin{aligned} {\begin{matrix} & \textbf{p} \sim \textrm{Dirichlet}([5,1,1,5]) \\ & p_{\text {rep}} \sim \textrm{uniform}(0, 1) \\ & \boldsymbol{\epsilon } {\mathop {\sim }\limits ^{iid}} \textrm{uniform}(0,.50), \\ \end{matrix}} \end{aligned}$$

where $\textbf{p}$ is the probability distribution over true preference states.

### Results

#### Model Comparison

We used Bayes factors to compare $\mathcal {M}_{\textrm{RPPH}}$ and $\mathcal {M}_{\textrm{DI}}$. One challenge with using Bayes factors is computing the integral in Eq. 12, which often lacks a closed-form solution and might be high dimensional. We used an efficient variant of importance sampling called stepping stone sampling (Xie et al., 2011), which is implemented in the Pigeons.jl package (Syed et al., 2022). Prior research has shown that stepping stone sampling is more efficient and more accurate at scale (Xie et al., 2011).

The model comparison results are summarized in Table 1. Across all trials, the $\log _{10}$ Bayes factors are strongly negative, indicating a high degree of support for $\mathcal {M}_{\textrm{DI}}$ over $\mathcal {M}_{\textrm{RPPH}}$. One potential issue with Bayes factors is that they can be sensitive to the choice of prior over parameters (Kass & Raftery, 1995). To address this concern, we performed an alternative analysis in which the prior distribution over preference states for $\mathcal {M}_{\textrm{DI}}$ was uniform: $\textbf{p} \sim \textrm{Dirichlet}(\textbf{1}_4)$, where $\boldsymbol{1}_{4}$ is a $4\times 1$ vector of ones. The results of this analysis were highly similar to those of the original analysis.

#### Posterior Predictive Distributions

**Fig. 4:**
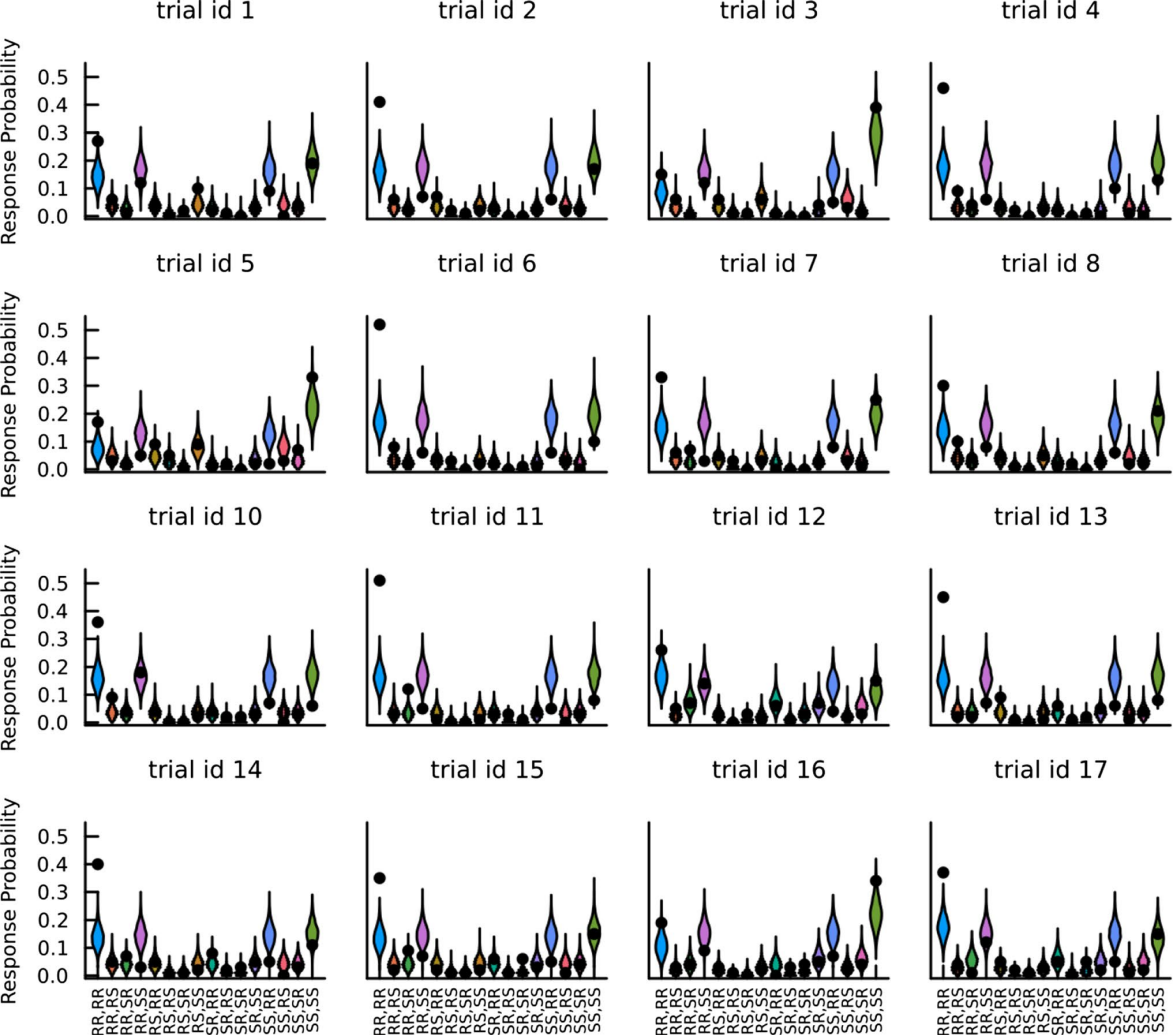
Posterior predictive distributions for $\mathcal {M}_{\textrm{RPPH}}$ for each trial. The posterior predictive response probabilities are represented as violin plots and mean empirical response probabilities are represented as black circles. Response patterns are labeled on the x-axis of the bottom row

We evaluated the posterior predictive distributions for each trial in Fig. 4 to gain insight into the failures of $\mathcal {M}_{\textrm{RPPH}}$. We used the No U-Turn sampler due to its ability to sample from the posterior distribution efficiently (Hoffman et al., 2014). Overall, there are large discrepancies between $\mathcal {M}_{\text {RPPH}}$ and the empirical data for most of the trials, with trials 3 and 12 having the smallest discrepancies. Trial 6 in Fig. 4 is emblematic of a systematic failure of the $\mathcal {M}_{\textrm{RPPH}}$: it underestimates $\Pr (R_p,R_f,R_p,R_f)$ in the left-most position and overestimates $\Pr (S_p,S_f,S_p,S_f)$ in the right-most position. In addition, the model overestimates the probability of semi-consistent response probabilities $\Pr (R_p,R_f,S_p,S_f)$ and $\Pr (S_p,S_f,R_p,R_f)$. Visual inspection of response probabilities $\Pr (R_p,R_f,R_p,R_f)$ and $\Pr (S_p,S_f,S_p,S_f)$ suggests that this systematic pattern of failures might be due to a violation of the critical inequality in Expression 15. We test this hypothesis more directly in the next section.

#### Critical Inequality

One straightforward approach for testing the critical inequality is to estimate a multinomial model for the 16 joint response categories and compare the parameters of the two response categories under investigation. According to this model, the data are distributed as $\textbf{Y} \sim \textrm{multinomial}(\boldsymbol{\theta }, n)$, with $\boldsymbol{\theta }$ being a $16 \times 1$ unit simplex representing category probabilities and *n* being the number of subjects. We use a uniform prior over the joint response probabilities:

16 $$\begin{aligned} \boldsymbol{\theta } \sim \textrm{Dirichlet}(\boldsymbol{1}_{16}). \end{aligned}$$

Next, we form the following variable to test the critical inequality:

17 $$\begin{aligned} \theta _{\text {diff}} | \textbf{y} = \theta _{1}|\textbf{y } - \theta _{16}|\textbf{y}, \end{aligned}$$

where $\theta _{1}|\textbf{y}$ is a posterior sample for the probability of the joint response category $R_p,R_f,R_p,R_f$, and $\theta _{16}|\textbf{y}$ is a posterior sample for the joint response category $S_p,S_f,S_p,S_f$.

**Table 2:** Tests of the critical inequality of the TEDM variant $\mathcal {M}_{\text {RPPH}}$ with $p_{S_pS_f} = 1$

trial id	HDI_95%_	trial id	HDI_95%_
1	$(-0.051, 0.184)$	8	$(-0.039, 0.2)$
2	$\mathbf {(0.091, 0.337)}$	10	$\mathbf {(0.168, 0.363) }$
3	$(-0.325, -0.084)$	11	$\mathbf {(0.268, 0.486)}$
4	$\mathbf {(0.168, 0.409)}$	13	$\mathbf {(0.203, 0.426)}$
5	$(-0.252, -0.019)$	14	$\mathbf {(0.139, 0.366)}$
6	$\mathbf {(0.237, 0.48)}$	15	$\mathbf {(0.05, 0.29) }$
7	$(-0.062, 0.192)$		

*HDI*_95%_ 95% highest density interval for $\theta _{\text {diff}} | \textbf{y}$ defined in Eq. 17. Bold entries indicate evidence against the critical inequality

We used the No U-turn sampler to perform the tests of the critical inequality specified in Inequality 15. Table 2 shows the results for the tests of the critical inequality. In eight of the 13 applicable cases, the prediction of the critical inequality is violated. In several cases, including trials 11 13, the violations are large and obvious based on visual inspection of Fig. 4. Overall, these results provide strong evidence against $\mathcal {M}_{\textrm{RPPH}}$.

### Discussion

Our goal in this first analysis was to test the ability of the PH to generalize from simple, single-stage gambles to dynamic inconsistency in two-stage gambles. In service of this goal, we extended the PH by incorporating a reference point dependent valuation process, resulting in a model we called the RPPH. To analyze the deterministic predictions of the RPPH, we used a TEDM to disentangle true preferences from response errors and response dependencies. Our analysis indicates that, although the RPPH can produce dynamic inconsistency under some conditions, its predictions are frequently at odds with empirical data patterns. For the majority of trials, the RPPH predicted preference for the safe option in the planned and final decision conditions, causing it to systematically underestimate risk seeking behavior. Perhaps the strongest evidence against the RPPH are the numerous violations of its critical inequality. What makes these results compelling is that they hold regardless of the parameter values used in the TEDM.

Although we used a TEDM to disentangle true preferences from various sources of errors and response dependencies, a critic could argue that the RPPH is overly simplistic because it fails to account for individual differences in error rates and aspiration levels. According to this argument, the basic structure of the RPPH could adequately describe decision making in the two-stage decision task, but failing to account for individual differences could lead to deceptively poor model performance. In the next analysis, we address this criticism by incorporating individual differences into the RPPH and comparing it to two existing models of the two-stage decision task—one based on prospect theory (Barkan & Busemeyer, 2003), and another based on quantum cognition (Busemeyer et al., 2015).

**Table 3:** A summary of the mechanisms used by each model to explain key phenomena

Phenomenon	QCM	*RPPT*	*SRPPH*
Response Dependencies	remember and repeat $p_{\textrm{rep}}$	remember and repeat $p_{\textrm{rep}}$	remember and repeat $p_{\textrm{rep}}$
Valuation	utility function risk aversion $\alpha$, loss aversion $\lambda$	utility function risk aversion $\alpha$, loss aversion $\lambda$	serial feature comparison aspiration levels $\delta$, decisional noise $\sigma$
Dynamic Inconsistency	quantum dynamics	reference point dependence	reference point dependence

*SRPPH* stochastic reference point priority heuristic, *RPPT* reference point prospect theory

## Analysis 2

In the second analysis, we test an extension of the RPPH which incorporates individual differences in aspiration levels and decisional noise. We term this model the Stochastic Reference Point Priority Heuristic (SRPPH) and analyze it at the level of individual subjects. The SRPPH builds upon an earlier generalization of the PH described in Rieskamp (2008). We also compare the SRPPH to two existing models in the literature: the Reference Point Prospect Theory (RPPT) (Barkan and Busemeyer, 2003), and a quantum cognition model (QCM) (Busemeyer et al., 2015). Table 3 summarizes the key model mechanisms under comparison. Each of the three models handles response dependencies in an identical manner. For this reason, differences in model performance cannot be attributed to this component. The models differ in their proposed valuation mechanisms. The valuation mechanism for the SRPPH is based on the sequential, non-compensatory process illustrated in Fig. 1. By contrast, the valuation mechanism for the RPPT and QCM integrates features in a compensatory fashion based on the utility function in prospect theory (Tversky & Kahneman, 1992). Differences between the SRPPH and the RPPT and the QCM can be attributed, at least in part, to the valuation mechanisms. Finally, the models use different mechanisms to produce the DI. Whereas DI arises from reference point dependence for SRPPH and RPPT, DI arises from uncertainty and entanglement in the QCM. Thus, differences between the QCM and the SRPPH and RPPT can be attributed, in part, to the reference point dependence and uncertainty and entanglement.

### Reference Point Prospect Theory

Reference point prospect theory (RPPT), also known as the reference point model (Tversky & Shafir, 1992; Barkan & Busemeyer, 2003), integrates features in a compensatory fashion using reference point dependence and the utility function based on prospect theory. In prospect theory, the utility function is non-linear and potentially weights losses more than gains (Tversky & Kahneman, 1992). Specifically, the utility function for outcome *x* is given by:

18 $$\begin{aligned} u(x) = {\left\{ \begin{array}{ll} x^\alpha \text { if } x \ge 0 \\ -\lambda |x|^\alpha \text { if } x < 0 \end{array}\right. }, \end{aligned}$$

where parameter $\alpha \ge 0$ represents risk aversion and $\lambda> 1$ represents loss aversion. Risk aversion occurs when $0 \le \alpha < 1$; risk neutrality occurs when $\alpha = 1$; risk seeking occurs when $\alpha> 1$. Loss aversion occurs when $\lambda> 1$, indicating that losses have more psychological impact than gains of equivalent magnitude.

In all three conditions, a decision is made by comparing the expected utility of selecting the risky option to the expected utility of the safe option. We will index the full set of conditions with $$k \in \{P,G,L\}$$, corresponding the planned condition, the final condition given a gain, and the final condition given a loss. Beginning with the *planned* condition, the outcome $$x_i$$ from stage 1 is not incorporated into the utility function. Following (Busemeyer et al., 2015), we assume that each outcome is equally weighted (i.e., $$w_G = w_L =.50)$$, resulting in the following expected utility for the risky option:

19 $$\begin{aligned} \text {EU}(\mathcal {R}) = \frac{1}{2} \left[ u(x_G) + u(x_L) \right] \end{aligned}$$

Similarly,the expected utility of selecting the safe option is given by:

20 $$\begin{aligned} \text {EU}(\mathcal {S}) = u(0) = 0. \end{aligned}$$

In the planned condition, the difference in expected utilities is denoted

21 $$\begin{aligned} d_P = \text {EU}(\mathcal {R}) - \text {EU}(\mathcal {S}). \end{aligned}$$

In the two *final* conditions, the experienced outcome from the first stage $x_i$, with condition $i \in \{G,L\}$, becomes a reference point for the second stage decision. Accordingly, $x_i$ is incorporated into the utility function. Given that $x_i$ was observed in the first stage, the expected utility for the risky option is defined as:

22 $$\begin{aligned} \text {EU}(\mathcal {R} \mid x_i) = \frac{1}{2} \left[ u(x_i + x_G) + u(x_i + x_L) \right] . \end{aligned}$$

Similarly, given that $x_i$ was observed in the first stage, the expected utility for the safe option is defined as:

23 $$\begin{aligned} \text {EU}(\mathcal {S} \mid x_i) = u(x_i + 0) = u(x_i). \end{aligned}$$

The difference in expected utilities is denoted:

24 $$\begin{aligned} d_i = \text {EU}(\mathcal {R} \mid x_i) - \text {EU}(\mathcal {S} \mid x_i) . \end{aligned}$$

For each condition *k*, the probability of selecting the risky option over the safe option is found by mapping the utility difference to a probability via the logistic function:

25 $$\begin{aligned} \Pr (R \mid k) = \frac{1}{1 + e^{-\eta d_k}}, \end{aligned}$$

where $\eta \ge 0$ controls the how sensitive the logistic function is to changes in the difference of expected utilities. When $\eta = 0$, the risky and safe options have equal selection probabilities. As $\eta$ increases, the choice behavior becomes increasingly deterministic. The complimentary probability of selecting the safe option is $\Pr (S \mid k) = 1 - \Pr (R \mid k)$

### Stochastic Reference Point Priority Heuristic

In this section, we introduce a variant of the RPPH based on the extension of the PH by Rieskamp (2008) which allows for individual variation in the aspiration levels and decisional noise. We will term this model the stochastic reference point priority heuristic (*SRPPH*). Using a Thurstonian approach (Thurstone, 1927), the key change in this model is that each feature difference between gamble $\mathcal {R}$ and gamble $\mathcal {S}$ is treated as a normally distributed random variable which is compared to an aspiration level. If the random variable is greater than the the aspiration level $\delta$, the risky option is selected. If the random variable is less than $-\delta$, the safe option is selected. If the random variable lies between $-\delta$ and $\delta$, the next feature is evaluated. To formulate the model’s choice probabilities, we first define the probabilities of events occurring at each feature evaluation stage. Once the decision probabilities for each feature evaluation stage are defined, we combine these terms to obtain the marginal probability of selecting the risky option.

The probability of selecting the risky option in the planned condition based on the minimum outcome is:

26 $$\begin{aligned} \Pr _{\text {min}}(R \mid P) = \Phi \left( \frac{\mu _{\text {min}} - \delta _{\text {min}}^{\prime }}{\sigma _{\text {min}}} \right) , \end{aligned}$$

where $\Phi (\cdot )$ is the standard normal cumulative distribution function, $\mu _{\text {min}} = h_{\text {min}}(\textbf{x}_R) - h_{\text {min}}(\textbf{x}_S)$ is the difference in minimum outcomes, $h_{\text {min}}$ is defined in Eq. 1, $\delta ^{\prime } = \delta _{\text {min}} \cdot x_{\text {max}}$ is the aspiration level, such that $0< \delta _{\text {min}} < 1$, and is the standard deviation of the minimum outcome. The probability of selecting the safe option is defined as:

27 $$\begin{aligned} \Pr _{\text {min}}(S \mid P) = 1 - \Phi \left( \frac{\mu _{\text {min}} + \delta _{\text {min}}^{\prime }}{\sigma _{\text {min}}} \right) . \end{aligned}$$

Accordingly, the probability of comparing the minimum outcome probabilities is $1 - \Pr _{\text {min}}(R \mid P) - \Pr _{\text {min}}(S \mid P)$. The probability of selecting the risky option in the planned condition when evaluating the minimum outcome probability is defined as:

28 $$\begin{aligned} \Pr _{\text {pr}}(R \mid P) = \Phi\left( \frac{\mu _{\text {pr}} - \delta _{\text {pr}}}{\sigma _{\text {pr}}} \right) _{-1}^{1} , \end{aligned}$$

where $\mu _{\text {pr}} = h_{\text {pr}}(\textbf{p}_S) - h_{\text {pr}}(\textbf{p}_R)$ is the difference in minimum outcome probabilities, $h_{\text {pr}}$ as defined in Equation 2, and $\Phi(\cdot )_{-1}^{1}$ is the normal cumulative distribution function which has been truncated between $-1$ and 1 to be valid for the difference between two probabilities. The probability of choosing the safe option in the planned condition when evaluating the minimum outcome probability is given by:

29 $$\begin{aligned} \Pr _{\text {pr}}(S \mid P) = 1 - \Phi \left( \frac{\mu _{\text {pr}} + \delta _{\text {pr}}}{\sigma _{\text {pr}}} \right)_{-1}^{1} . \end{aligned}$$

When evaluating the maximum outcomes, the difference is compared to zero instead of an aspiration level based on $\delta$. The probability of selecting the risky option in the planned condition when evaluating the maximum outcome is defined as:

30 $$\begin{aligned} \Pr _{\text {max}}(R \mid P) = \Phi \left( \frac{\mu _{\text {max}}}{\sigma _{\text {max}}} \right) , \end{aligned}$$

where $\mu _{\text {max}} = h_{\text {max}}(\textbf{x}_R) - h_{\text {max}}(\textbf{x}_S)$ is the difference in maximum outcomes, and $h_{\text {max}}$ is defined in Equation 3. In this case, the probability of selecting the safe option is $1 - \Pr _{\text {max}}(R \mid P)$. Following Rieskamp (2008), we reduce the model to a single noise parameter $\sigma$ by applying the following constraints: $\sigma _{\text {pr}} = \sigma$ and $\sigma _{\text {min}} = \sigma _{\text {max}} = \sigma \cdot x_{\text {max}}$.

The probability of selecting the risky option is found by summing the probabilities of the three ways in which the risky option can be selected:

31 $$\begin{aligned} \begin{aligned} \Pr (R \mid P) =&\\ &\Pr _{\text {min}}(R \mid P) + \\ &\left[ 1 - \Pr _{\text {min}}(R \mid P) - \Pr _{\text {min}}(S \mid P)\right] \cdot \Pr _{\text {pr}}(R \mid P) + \\&\left[ 1 - \Pr _{\text {min}}(R \mid P) - \Pr _{\text {min}}(S \mid P)\right] \cdot \\ &\left[ 1 - \Pr _{\text {pr}}(R \mid P) - \Pr _{\text {pr}}(S \mid P)\right] \cdot \Pr _{\text {max}}(R \mid P). \end{aligned} \end{aligned}$$

The equations above can be easily modified to form the corresponding probabilities in the final condition. This process involves adding the observed outcome from the first stage $x_i$ to the inputs for $h_{\text {min}}$ and $h_{\text {max}}$.

### Quantum Cognition Model

We compared the *SRPPH* to a Quantum Cognition Model (QCM) of dynamic inconsistency developed by Busemeyer et al. (2015). In the QCM, DI arises from the interaction between the quantum dynamics of the decision process and uncertainty in the outcome from the first stage (Busemeyer et al., 2015). The QMC has a similar structure to other quantum models of cognition, including one developed for the disjunction effect in the prisoner’s dilemma (Pothos & Busemeyer, 2009) and another developed for interference effects between categorization and decision making (Wang & Busemeyer, 2016). A key difference between the QCM and many other models is that its probabilistic foundation is based on quantum probability theory rather than classical probability theory. Compared to the more familiar classical probability theory, quantum probability theory is based on a different set of axioms, and can be conceptualized as a generalization of classical probability theory in which probabilities are not required to commute, e.g., $\Pr (a, b) \ne \Pr (b,a)$, and the law of total probability need not hold, e.g., $\Pr (a) \ne \Pr (a, b) + \Pr (a, \lnot b)$ (Busemeyer et al., 2011).

**Table 4:** Prior distributions used for Bayesian parameter estimation and Bayesian model comparison

Moderately Informed Priors		
*SRPPH*	QCM	*RPPT*
$\sigma \sim \text {normal}(1,2)_{0}^{\infty }$	$\alpha \sim \text {normal}(1,1)_{0}^3$	$\alpha \sim \text {normal}(1,1)_{0}^3$
$\delta _{\text {min}} \sim \text {beta}(1,9)$	$\lambda \sim \text {normal}(1,2)_{1}^6$	$\lambda \sim \text {normal}(1,2)_{1}^6$
$\delta _{\text {prob}} \sim \text {beta}(1,9)$	$\gamma \sim \text {normal}(0,10)$	$\eta \sim \text {normal}(1, .5)_{0}^2$
$p_{\textrm{rep}} \sim \text {uniform}(0, 1)$	$p_{\textrm{rep}} \sim \text {uniform}(0, 1)$	$p_{\textrm{rep}} \sim \text {uniform}(0, 1)$
Uninformed Priors		
*SRPPH*	QCM	*RPPT*
$\sigma \sim \text {uniform}(0,10)$	$\alpha \sim \text {uniform}(0,3)$	$\alpha \sim \text {uniform}(0,3)$
$\delta _{\text {min}} \sim \text {uniform}(0,1)$	$\lambda \sim \text {uniform}(1,6)$	$\lambda \sim \text {uniform}(1,6)$
$\delta _{\text {prob}} \sim \text {uniform}(0,1)$	$\gamma \sim \text {uniform}(-20,20)$	$\eta \sim \text {uniform}(0,2)$
$p_{\textrm{rep}} \sim \text {uniform}(0,1)$	$p_{\textrm{rep}} \sim \text {uniform}(0,1)$	$p_{\textrm{rep}} \sim \text {uniform}(0,1)$

Classical probability theory represents events as sets using Boolean logic. However, quantum probability theory represents events geometrically as subspaces within a type of vector space called a Hilbert space (Busemeyer et al., 2011; Busemeyer & Bruza, 2012). The QCM represents the four combinations of first stage outcomes and second stage decisions with the following orthonormal basis vectors: $$|\textrm{GR}\rangle = \left[ 1, 0, 0, 0\right] ^\top$$, $$|\textrm{GS}\rangle = \left[ 0, 1, 0, 0\right] ^\top$$, $$|\textrm{LR}\rangle = \left[ 0, 0, 1, 0\right] ^\top$$, and $$|\textrm{LS}\rangle = \left[ 0, 0, 0, 1\right] ^\top$$, where G and L correspond to a gain and a loss in the first stage and *R*, and *S* correspond to selecting the risky and safe options in the second stage. A person’s cognitive state is represented as a superposition state vector, denoted $$|\psi \rangle = \psi _{\textrm{GR}} \cdot |\textrm{GR}\rangle +\psi _{\textrm{GS}}\cdot |\textrm{GS}\rangle$$$$\psi _{\textrm{LR}} \cdot |\textrm{LR}\rangle + \psi _{\textrm{LS}}\cdot |\textrm{LS}\rangle$$, which is a linear combination of the orthonormal basis vectors. Elements in $$|\psi \rangle$$ represent amplitudes, or probabilities when squared. The probability of an event is found by projecting $$|\psi \rangle$$ on to the corresponding subspace and squaring its magnitude.

Before observing an outcome from the first stage, a person is in the following superposition state:

32 $$\begin{aligned} |\mathbf {\psi }_0\rangle = \left[ \frac{1}{2},\frac{1}{2},\frac{1}{2},\frac{1}{2}\right] ^\top , \end{aligned}$$

indicating all combinations of outcomes and decisions are equally likely. In the final decision condition, the superposition state is updated upon observing $x_G$ or $x_L$ in the first stage. If $x_G$ is observed, the amplitudes corresponding to a loss are set to zero, and the superposition state is re-normalized as follows:

33 $$\begin{aligned} |\mathbf {\psi }_G\rangle = \frac{1}{\sqrt{2}}\left[ 1,1,0,0\right] ^\top . \end{aligned}$$

The updated superposition state after observing $x_L$ is defined similarly:

34 $$\begin{aligned} |\mathbf {\psi }_L\rangle = \frac{1}{\sqrt{2}}\left[ 0,0,1,1\right] ^\top . \end{aligned}$$

The decision process evolves as a quantum wave form according to Schrödinger’s equation, which represents momentary vacillations between the risky and safe options. The solution to Schrödinger’s equation yields the unitary matrix, defined as:

$$\begin{aligned} \textbf{U} = e^{-i \cdot \frac{\pi }{2} \cdot \textbf{H}}, \end{aligned}$$

where $\textbf{H}$ is the Hamiltonian matrix. The Hamiltonian matrix can be separated into the sum of two component matrices: $\textbf{H} = \textbf{H}_1 + \textbf{H}_2$. The first component matrix is:

$$\begin{aligned} \textbf{H}_{1} = \begin{bmatrix} \frac{\upsilon _{G}}{\sqrt{1 + \upsilon _{G}^2}} & \frac{1}{\sqrt{1 + \upsilon _{G}^2}} & 0 & 0\\ \frac{1}{\sqrt{1 + \upsilon _{G}^2}} & \frac{-\upsilon _{G}}{\sqrt{1 + \upsilon _{G}^2}} & 0 & 0\\ 0 & 0 & \frac{\upsilon _{L}}{\sqrt{1 + \upsilon _{L}^2}} & \frac{1}{\sqrt{1 + \upsilon _{L}^2}}\\ 0 & 0 & \frac{1}{\sqrt{1 + \upsilon _{L}^2}} & \frac{-\upsilon _{L}}{\sqrt{1 + \upsilon _{L}^2}} \\ \end{bmatrix}, \end{aligned}$$

where $\upsilon _i = \text {tanh}(.5 \cdot d_i)$, and the utility difference $d_i$ is identical to that used in the *RPPT* model as defined in Eqs. 21 and 24. The second component matrix is:

$$\begin{aligned} \textbf{H}_{2} = \frac{-\gamma }{\sqrt{2}}\begin{bmatrix} 1 & 0 & 1 & 0\\ 0 & -1 & 0 & 1\\ 1 & 0 & -1 & 0\\ 0 & 1 & 0 & 1\\ \end{bmatrix}, \end{aligned}$$

where $\gamma$ is the entanglement parameter, which controls the degree of interference. The projection matrix for selecting the risky option is given by:

$$\begin{aligned} \textbf{M} = \begin{bmatrix} 1 & 0 & 0 & 0\\ 0 & 0 & 0 & 0\\ 0 & 0 & 1 & 0\\ 0 & 0 & 0 & 0\\ \end{bmatrix}. \end{aligned}$$

This matrix projects the superposition state onto the sub-space spanned by basis vectors $|\text {GR}\rangle$ and $|\text {LR}\rangle$, i.e., the basis vectors associated with selecting the risky option. With these quantities defined, the probability of selecting the risky option in the planned condition is given by:

35 $$\begin{aligned} \Pr (R \mid P) = \Vert \textbf{M} \cdot \textbf{U} \cdot |\mathbf {\psi }_0\rangle \Vert ^2. \end{aligned}$$

In the final condition, the probability of selecting the risky option after observing $x_G$ is:

36 $$\begin{aligned} \Pr (R \mid G) = \Vert \textbf{M} \cdot \textbf{U} \cdot |\mathbf {\psi }_G\rangle \Vert ^2 \end{aligned}$$

Similarly, in the final condition, the probability of selecting the risky option after observing $x_L$ is:

37 $$\begin{aligned} \Pr (R \mid L) = \Vert \textbf{M} \cdot \textbf{U} \cdot |\mathbf {\psi }_L\rangle \Vert ^2. \end{aligned}$$

**Fig. 5:**
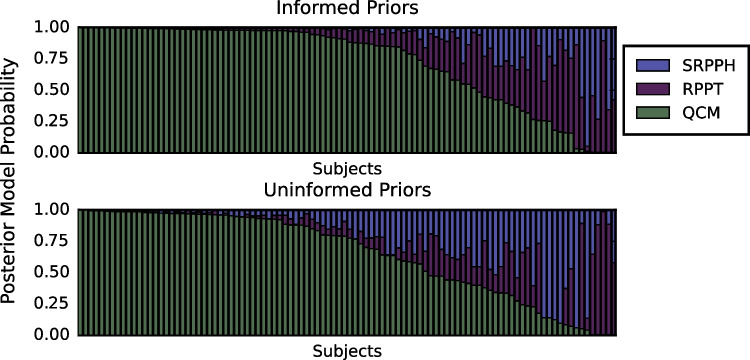
*Top* posterior model probabilities for informed priors. *Bottom* posterior model probabilities for uninformed priors. Model priors are assumed to be equal in each case. Each triplet of vertically stacked bars represents the posterior model probability for a single subject, which sums to 1. Subjects are sorted in descending order according to posterior probability of the quantum model. *QCM* quantum model. *SRPPH* stochastic reference point priority heuristic. *RPPT* reference point prospect theory

An important property the QCM is how uncertainty changes the evolution of the decision. Uncertainty in the first stage outcome in the planned decision condition creates interference, leading to DI. This can be seen in the violation of the law of total probability which holds that $$\Pr (R \mid P) = \Pr (R \mid G) \cdot$$$$\Pr (G) + \Pr (R \mid L) \cdot \Pr (L)$$ (Busemeyer et al., 2015). In other words, the probability of selecting the risky option when the first stage outcome is unknown is a mixture of the probabilities when the outcome is a gain or a loss. However, in the QCM, the law of total probability does not necessarily hold because uncertainty about the first stage outcome in the planned decision condition creates interference: $$\Pr (R \mid P) = \Pr (R \mid G)\frac{1}{2} + \Pr (R \mid L)\frac{1}{2} + \text {int}$$ (Busemeyer et al., 2015). The term int, which can be positive or negative, represents interference. The entanglement parameter $$\gamma$$ introduced above modulates the degree of interference in the QCM.

### Log Likelihood Function

The same multinomial log likelihood function is used in each of the three models to define joint log probability of choices in the *planned* and corresponding *final* condition. Letting *g* index the trial, $j \in \{S_p,R_p\}$ index the responses in the planned decision condition, and $m \in \{S_f,R_f\}$ index the responses in the final decision condition, then $\log (\Pr _{g}(j,m))$ is the log of the joint response probability and $y_{g,j,m}$ is the observed joint response frequency. The log likelihood can be found by summing across the joint probabilities for each trial:

38 $$\begin{aligned} \text {LL} = \sum _{g=1}^{33} \sum _{j,m} y_{g,j,m} \cdot \log (\Pr _{g}(j,m)). \end{aligned}$$

The joint probabilities below consist of two components: the model specific choice probabilities, and dependency between responses in the *planned* and corresponding *final* final condition for the same trial.

39 $$\begin{aligned} \begin{aligned} \Pr (R_p,R_f)&= \Pr (R \mid P) \cdot \left[ p_{\text {rep}} + (1 - p_{\text {rep}}) \cdot \Pr (R \mid i) \right]&\\ \Pr (R_p,S_f)&= \Pr (R \mid P) \cdot (1 - p_{\text {rep}}) \cdot \Pr (S \mid i)&\\ \Pr (S_p,R_f)&= \Pr (S \mid P) \cdot (1 - p_{\text {rep}}) \cdot \Pr (R \mid i)&\\ \Pr (S_p,S_f)&= \Pr (S \mid P) \cdot \left[ p_{\text {rep}} + (1 - p_{\text {rep}}) \cdot \Pr (S \mid i) \right]&\\ \end{aligned} \end{aligned}$$

where $i \in \{G, L\}$. Note that the trial index *g* is suppressed.

## Results

As shown in Table 4, we used two sets of priors to assess the sensitivity of the model comparison to the choice of prior distribution over model parameters. The moderately informed priors placed more density on likely parameter values when applicable. For example, in the *SRPPH*, prior distribution for the aspirational level parameters ($\delta$) were $\textrm{beta}(1,9)$, which has a mean equal to the theoretical value of.10 assumed in the PH and a standard deviation of .09. For the uniformed priors, we used uniform priors. In many cases, the uniform priors were truncated in order to avoid numerical overflow occurring with extreme values. To ensure valid comparisons, we used identical priors for models sharing the same parameters.

**Fig. 6:**
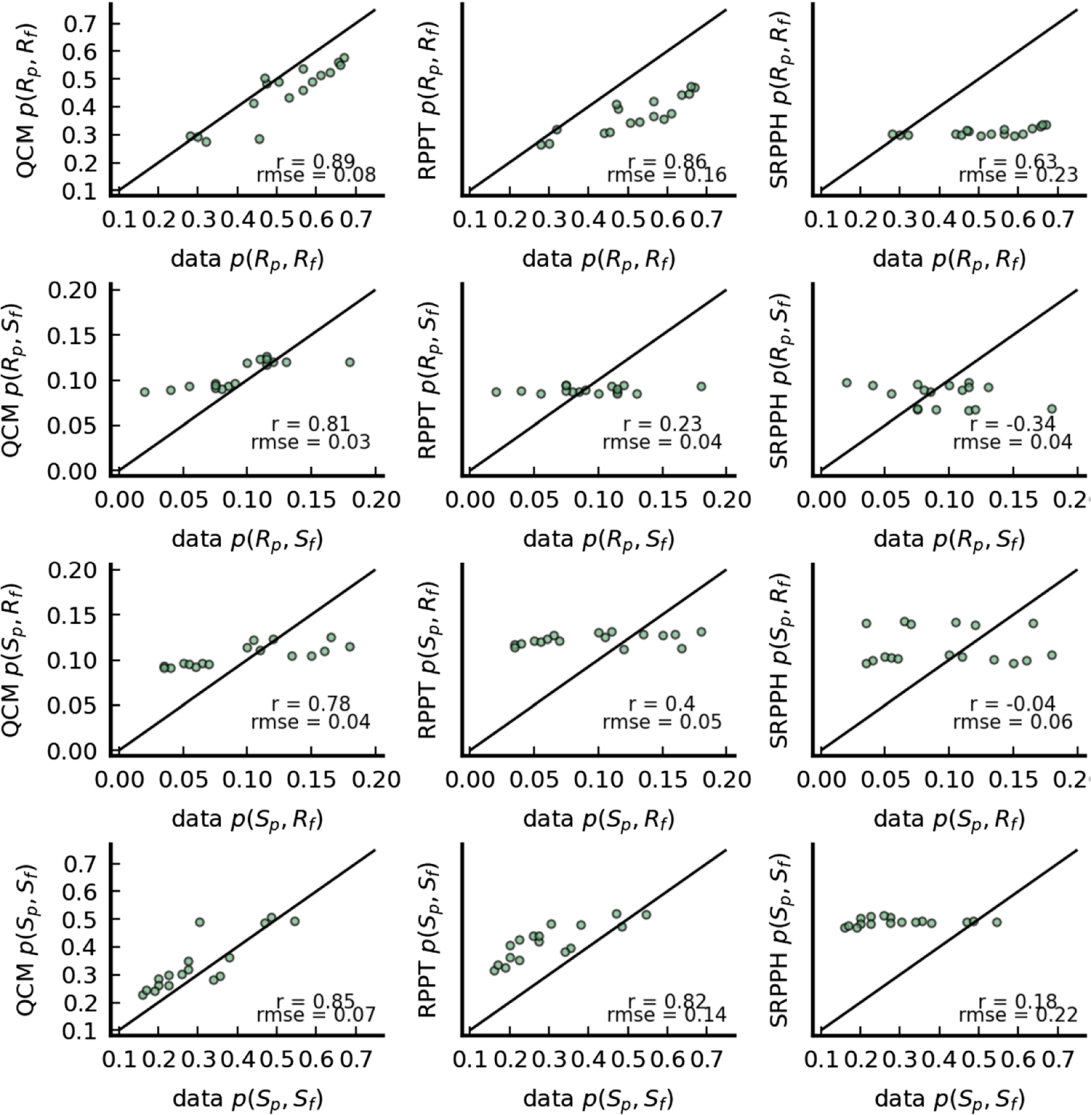
Posterior predictive distribution of joint probabilities (rows) for each model (columns). The x-axis represents the mean response probability across subjects. The y-axis represents the mean of the posterior predictive distribution. Each enclosed circle represents a unique trial. Correlation and root mean squared error (RMSE) are located in the bottom right of each subplot

### Model Comparison

We used the stepping stone sampling algorithm (Xie et al., 2011; Syed et al., 2022) to estimate the parameters and marginal log likelihood of each individual for each model. Unlike many MCMC sampling algorithms, the stepping stone sampling algorithm is compatible with imaginary numbers and multi-modal posterior distributions commonly found with quantum cognition models. We used posterior model probabilities rather than Bayes factors given that there are more than two models under consideration. The posterior model probability for $\mathcal {M}_i$ is defined as:

40 $$\begin{aligned} p(\mathcal {M}_i \mid \textbf{Y}) = \frac{f(\textbf{Y} \mid \mathcal {M}_i)p(\mathcal {M}_i)}{\sum _{\mathcal {M}_j \in \boldsymbol{\mathcal {M}}} f(\textbf{Y} \mid \mathcal {M}_j)p(\mathcal {M}_j)}, \end{aligned}$$

and can be computed from the marginal log likelihood values obtained through stepping stone sampling. In the results reported below, we set the model priors to be equal.

The model comparison results are summarized by individual subject in Fig. 5. Overall, the posterior model probability was the greatest for the majority of subjects for the QCM regardless of priors used for model parameters. When using moderately informed priors, the proportion of subjects with the highest posterior probability was $82\%$ for the QCM, $11\%$ for the *RPPT* and $7\%$ for the *SRPPH*. When using uninformed priors, the proportion of subjects with the highest posterior probability was $74\%$ for the QCM, $15\%$ for the *SRPPH* and $11\%$ for the *RPPT*.

#### Posterior Predictive Distributions

We compared the posterior predictive distributions to the observed data to identify model deficiencies. Our basic approach was to compare the empirical data and posterior predictions in terms of the joint response probabilities of each trial averaged across subjects, allowing us to determine whether the models captured response patterns across trials at the group level.

The mean of the posterior predictive distributions are summarized in Fig. 6 as a grid of scatter plots comparing the predictions to the data for each model and each joint response. Overall, the QCM provided the best account of the data, followed by the *RPPT* and then the *SRPPH*. All models underestimated the variance in response probabilities across trials, but the degree of underestimation was less for the QCM compared to the other models. In general, the QCM captured the variation in response probabilities across trials, yielding high correlations and low RMSEs. The main deficiency of the QCM is that it underestimated variance in responses probabilities $\Pr (S_{p},R_{f})$ and $\Pr (R_{p},S_{f})$ across trials. There were three main results for the *RPPT*. First, the correlation between the predictions and the data were high for $\Pr (R_{p},R_{f})$ and $\Pr (S_{p},S_{f})$. Second, the *RPPT* underestimated $\Pr (R_{p},R_{f})$ and overestimated $\Pr (S_{p},S_{f})$. Third, the *RPPT* failed to adequately capture variation in dynamic inconsistency across trials (see response categories $\Pr (R_{p},S_{f})$ and $\Pr (S_{p},R_{f})$). Overall, the *SRPPH* performed poorly, as evidenced by the generally low correlations and high RMSE. Much like the *RPPT*, the *SRPPH* underestimated $\Pr (R_{p},R_{f})$ and overestimated $\Pr (S_{p},S_{f})$. It also failed to capture variation in dynamic inconsistency across trials.

### Discussion

In the second model analysis, we developed the *SRPPH*, a more general variant of the RPPH inspired by earlier work (Rieskamp, 2008), which allows individuals to differ in terms of aspiration level and decision noise and tested the it against two established models of DI—one based on quantum cognition (i.e., the QCM) and another based on prospect theory (i.e., the *RPPT*). Importantly, these models differ in terms of the valuation process (compensatory vs. non-compensatory) and the mechanism responsible for DI (reference point dependence vs. quantum dynamics), which allows us to make some inferences regarding our central question about compensatory and non-compensatory decision making.

We compared the models with two lines of evidence: posterior model probabilities at the individual level, and the ability of the models to capture variation across trials at the group-level. Looking across all of the evidence, we found the most support for the QCM, followed by the *RPPT* and the *SRPPH*. At the individual level, the data provided the most support for the QCM. Nonetheless, a sizable minority of individuals were better characterized by the *RPPT* or *SRPPH*. This finding could indicate that a one-size fits all model may not be viable, or perhaps a model we did not consider could provide a better characterization of DI. At the group level, the models also differed in their ability to capture variation across gambles at the group level. In this analysis, the data also supported the models in the following order: QCM, *RPPT* and *SRPPH*. To varying degrees, all three models underestimated the variance in joint choice probabilities, particularly for the DI. Although the QCM performed notably better in this regard, there may still be room for improvement.

Overall, the results of the second model analysis cast further doubt on the ability of the PH to generalize to two-stage decisions. Allowing individual variation in aspiration level and decisional noise does not sufficiently improve the model. The evidence points most strongly to a compensatory model with a decision process governed by quantum principles from quantum cognition.

## General Discussion

Our motivation for the current research was to contrast compensatory and non-compensatory accounts of decision making. In other words, do people evaluate all available information, or do they prioritize only a subset of the information available? Models based on expected utility theory, which are ubiquitous in many fields, posit that people evaluate options in a compensatory fashion by considering all features of options when making a decision. By contrast, fast and frugal heuristics, such as the PH, posit that people evaluate options in a non-compensatory fashion, focusing only on a subset of features. Our approach to testing these contrasting theoretical perspectives was to investigate the ability the PH generalize to a more complex two-stage decision making task in which DI is commonly observed. In service of this goal, we extended PH by incorporating a reference point dependent valuation process used in prior research, forming what we termed the RPPH.

Our first analysis of the RPPH revealed that it can produce dynamic inconsistency under some conditions. However, beyond the requisite ability to produce DI, the predictions of the RPPH were largely at odds with patterns in previously reported data, even after accounting for response errors and response dependencies with a TEM analysis. One particularly strong failure of the RPPH was the violation of its critical inequality between the joint probabilities of consistently selecting the safe options and consistently selecting the risky options. In this case, the evidence against the RPPH is notably strong because the RPPH cannot accommodate the data by changing the value of its parameters. The structure of the model is simply deficient.

In a second model analysis, we developed a variant of the RPPH called the *SRPPH* which allowed individuals to vary according to aspiration levels and decisional noise. We compared the *SRPPH* to two established models—one based on quantum cognition (QCM), and another based on prospect theory (*RPPT*). This model comparison was important because it allowed us to disentangle the valuation process (i.e., compensatory vs. non-compensatory decision making) from the mechanism used to produce DI. Overall, we found moderate support for *RPPT* compared to *SRPPH*, which suggests the compensatory expected utility valuation of prospect theory better supported by the data than the non-compensatory decision process of the PH. We also found strong support for the QCM compared to the *RPPT*. Given that both share the same utility function based on prospect theory, this evidence suggests that the quantum dynamics posited by QCM are more consistent with the data than the reference point dependence mechanism.

### Limitations

The conclusions drawn from our model analysis should be interpreted in consideration of several limitations. One limitation with our extension of the PH to two-stage decision making is that reference point dependence is not central to the PH. In its original form, the PH does not have a mechanism to produce DI. As such, we incorporated reference point dependence into the PH for two reasons: (1) it is a simple and plausible mechanism, and (2) it has been used successfully in previous models (Barkan & Busemeyer, 2003). Nonetheless, in principle, an alternative mechanism could be used which could lead to more accurate predictions.

Another potential limitation is whether PH is applicable to the choice sets used in Barkan and Busemeyer (2003). As originally defined in Brandstätter et al. (2006), the PH is applicable to difficult decisions for which the ratio of expected values of the two options is less than 2. The ratio of expected values is undefined in the planned decision condition because the minimum expected value is zero. However, we argued that these decisions were not easy according to other reasonable criteria. First, neither option in the choice sets were stochastically dominated by the other option. Second, the choices were difficult according to the signal-to-noise measure in Eq. 8. One benefit of this alternative measure of difficulty is that it is defined when either option has an expected value of zero. Additionally, it takes into account the variance of each option. Was there any evidence to suggest that the decisions were easy? One might expect an easy decision would lead subjects to converge on a single superior option. However, the observed variation in choice probability (see Figs. 4 and 6) does not support the notion that the decisions were easy. To fully dispel any doubt, however, future research may consider repeating the two-stage experiment with modified gambles that satisfy the ratio criterion for decision difficulty.

## Conclusions

Our findings add to a growing list of doubts concerning the PH as an account of decision making. We found that two variations of the PH generalized poorly from single stage decision tasks to two-stage decision tasks. Overall, the evidence favors the compensatory decision making and quantum dynamics embodied in the QCM.

## Data Availability

Data are available at https://github.com/itsdfish/dynamic_inconsistency_model_comparison

## Appendix A True and Error Model with Response Dependencies

In this appendix, we extend the True and Error Model (Birnbaum, 2013; Birnbaum, 2023) to account for possible dependencies between responses in the planned and final decision conditions within a repetition of the same gamble. We term the resulting model the True and Error Dependent Model (TEDM). Before introducing the TEDM, we provide a brief overview of the TEM as necessary background. After explaining the logic of the TEDM, we present the 16 equations that comprise the model.

### A.1 True and Error Model

In the two-stage decision task, subjects make consecutive decisions between a safe option $\mathcal {S}$ and a risky option $\mathcal {R}$ in the second stage under two different conditions. First, in the planned condition, subjects make the decision based on a *hypothetical* outcome from the first stage. Second, in the final condition, subjects make the same decision after experiencing a *real* outcome from the first stage. Trials are randomized using different gambles and repeated twice. The TEM is comprised of two types of parameters: a vector of preference state parameters $\textbf{p}$, and a vector of error parameters $\boldsymbol{\epsilon }$. The preference state parameters $\mathcal {P}{\mathrm {R_{p}R_{f}}}, p_{\mathrm {R_{p}S_{f}}}, p_{\mathrm {S_{p}R_{f}}}, p_{\mathrm {S_{p}S_{f}}} \ge 0$ are joint probabilities defined over the preference states $\mathcal {P} = \{S_{p}S_{f},S_{p}R_{f},R_{p}S_{f},R_{p}R_{f}\}$, where *S* represents preference for the safe option, *R* represents preference for the risky option, and indices *p* and *f* correspond to the planned and final decision conditions, respectively. Thus, the preference state probabilities are subject to the constraint $\sum _{j \in \mathcal {P}} p_j = 1$. The four error parameters are $0 \le \epsilon _{\textrm{S}_p},\epsilon _{\textrm{S}_f},\epsilon _{\textrm{R}_p},\epsilon _{\textrm{R}_f} \le .50$, which represent the probability of selecting an option one does not prefer as a result of error. The TEM consists of 16 equations representing the joint probabilities over planned and final responses in the two replications. As an example, consider the response pattern $R_p,R_f,R_p,S_f$. As defined in Eq. 14, the probability of this response pattern is a mixture over preference states $\mathcal {P}$. For simplicity, we will focus only on the term in the equation associated with preference state for the risky option in both the planned and final decision conditions:

A1 $$\begin{aligned} p_{\mathrm {R_{p}R_{f}}} \cdot (1 - \epsilon _{\textrm{S}_{p}}) \cdot (1 - \epsilon _{\textrm{S}_{f}}) \cdot (1 - \epsilon _{\textrm{S}_{p}}) \cdot \epsilon _{\textrm{S}_{f}} . \end{aligned}$$

We can breakdown Expression A1 as follows: a person truly prefers $R_p$ and $R_f$ with probability $p_{\mathrm {R_{p}R_{f}}}$, and in the first replication, a person selects the risky option in the planned condition according to his or preferences with probability $(1 - \epsilon _{\textrm{S}_p})$ and selects the risky option in the final decision conditions according to his or her preferences with probability $(1 - \epsilon _{\textrm{S}_f})$. In the second replication, a person selects the risky option in the planned condition according to his or preferences with probability $(1 - \epsilon _{\textrm{S}_p})$, but erroneously selects the safe option in the final condition with probability $\epsilon _{\textrm{S}_f}$. Importantly, the response probabilities are assumed to be independent in the TEM.

### A.2 True and Error Dependent Model

The TEDM is designed to account for the possibility of dependence between planned and final decisions within the same replication of a gamble. In the TEDM, we follow a similar approach used in other models to account for response dependencies (Busemeyer et al., 2015). This approach involves introducing a new parameter $p_{\text {rep}}$, which represents the probability of simply repeating the response from the planned condition as the response for the final condition (within the same replication) instead of independently proceeding through the posited decision process (e.g., the RPPH). When $p_{\text {rep}} = 0$, the TEDM reduces to the TEM, and when $p_{\text {rep}}> 0$, some degree of dependence exists between planned and final decisions within the same replication. In the TEDM, repeated responses within the same repetition (e.g.,$R_p,R_f$) arise from a mixture of two response processes: (1) simply repeating the first response with probability $p_{\text {rep}}$, and (2) arriving at the same response through the posited decision process with probability $(1 - p_{\text {rep}})$. If the responses differ within the same repetition, the response probabilities are multiplied by $(1 - p_{\text {rep}})$, reflecting the fact that the second response was not a simple repetition of the first response. Putting these concepts together, we obtain an extended version of Expression 41:

A2 $$\begin{aligned}&p_{R_{p}R_{f}} \cdot (1 - \epsilon _{\textrm{S}_p}) \cdot (p_{\text {rep}} + (1 - p_{\text {rep}}) \\&\cdot (1 - \epsilon _{\textrm{S}_f})) \cdot (1 - p_{\text {rep}}) \cdot (1 - \epsilon _{\textrm{S}_p}) \cdot \epsilon _{\textrm{S}_f}. \end{aligned}$$

By expanding Expression 42, the mixture components can be clearly presented on two separate lines:

A3 $$\begin{aligned} \begin{aligned}&p_{R_{p}R_{f}} \cdot (1 - \epsilon _{\textrm{S}_p}) \cdot p_{\text {rep}} \cdot (1 - \epsilon _{\textrm{S}_p}) \cdot (1 - p_{\text {rep}}) \cdot \epsilon _{\textrm{S}_f} + \\&p_{R_{p}R_{f}} \cdot (1 - \epsilon _{\textrm{S}_p}) \cdot (1 - p_{\text {rep}}) \cdot (1 - \epsilon _{\textrm{S}_f}) \cdot (1 - \epsilon _{\textrm{S}_p}) \cdot (1 - p_{\text {rep}}) \cdot \epsilon _{\textrm{S}_f}. \end{aligned} \end{aligned}$$

It is easy to show that Expression 43 simplifies to Expression 41 of the TEM when $p_{\text {rep}} = 0$: the term in the top line is equal to zero, and the term in the second line is equal to Expression 41 because $(1 - p_{\text {rep}}) = 1$. This same logic applies to the remaining equations of the TEDM, as presented below.

### A.3 TEDM Equations

The 16 equations below comprise the joint probabilities of the TEDM.

$$\begin{aligned} \Pr (R_p,R_f,R_p,R_f) = \ &p_{R_{p}R_{f}} \cdot (1 - \epsilon _{\textrm{S}_p}) \cdot (p_{\text {rep}} + (1 - p_{\text {rep}}) \cdot (1 - \epsilon _{\textrm{S}_f})) \cdot (1 - \epsilon _{\textrm{S}_p}) \cdot (p_{\text {rep}} + (1 - p_{\text {rep}}) \cdot (1 - \epsilon _{\textrm{S}_f})) + \\&p_{R_{p}S_{f}} \cdot (1 - \epsilon _{\textrm{S}_p}) \cdot (p_{\text {rep}} + (1 - p_{\text {rep}}) \cdot \epsilon _{\textrm{R}_f}) \cdot (1 - \epsilon _{\textrm{S}_p}) \cdot (p_{\text {rep}} + (1 - p_{\text {rep}}) \cdot \epsilon _{\textrm{R}_f}) + \\&p_{S_{p}R_{f}} \cdot \epsilon _{\textrm{R}_p} \cdot (p_{\text {rep}} + (1 - p_{\text {rep}}) \cdot (1 - \epsilon _{\textrm{S}_f})) \cdot \epsilon _{\textrm{R}_p} \cdot (p_{\text {rep}} + (1 - p_{\text {rep}}) \cdot (1 - \epsilon _{\textrm{S}_f})) + \\&p_{S_{p}S_{f}} \cdot \epsilon _{\textrm{R}_p} \cdot (p_{\text {rep}} + (1 - p_{\text {rep}}) \cdot \epsilon _{\textrm{R}_f}) \cdot \epsilon _{\textrm{R}_p} \cdot (p_{\text {rep}} + (1 - p_{\text {rep}}) \cdot \epsilon _{\textrm{R}_f}) \\ \Pr (R_p,R_f,R_p,S_f) = \ &p_{R_{p}R_{f}} \cdot (1 - \epsilon _{\textrm{S}_p}) \cdot (p_{\text {rep}} + (1 - p_{\text {rep}}) \cdot (1 - \epsilon _{\textrm{S}_f})) \cdot (1 - p_{\text {rep}}) \cdot (1 - \epsilon _{\textrm{S}_p}) \cdot \epsilon _{\textrm{S}_f} + \\&p_{R_{p}S_{f}} \cdot (1 - \epsilon _{\textrm{S}_p}) \cdot (p_{\text {rep}} + (1 - p_{\text {rep}}) \cdot \epsilon _{\textrm{R}_f}) \cdot (1 - p_{\text {rep}}) \cdot (1 - \epsilon _{\textrm{S}_p}) \cdot (1 - \epsilon _{\textrm{R}_f}) + \\&p_{S_{p}R_{f}} \cdot \epsilon _{\textrm{R}_p} \cdot (p_{\text {rep}} + (1 - p_{\text {rep}}) \cdot (1 - \epsilon _{\textrm{S}_f})) \cdot (1 - p_{\text {rep}}) \cdot \epsilon _{\textrm{R}_p} \cdot \epsilon _{\textrm{S}_f} +\\&p_{S_{p}S_{f}} \cdot \epsilon _{\textrm{R}_p} \cdot (p_{\text {rep}} + (1 - p_{\text {rep}}) \cdot \epsilon _{\textrm{R}_f}) \cdot (1 - p_{\text {rep}}) \cdot \epsilon _{\textrm{R}_p} \cdot (1 - \epsilon _{\textrm{R}_f})\\ \Pr (R_p,R_f,S_p,R_f) = \ &p_{R_{p}R_{f}} \cdot (1 - \epsilon _{\textrm{S}_p}) \cdot (p_{\text {rep}} + (1 - p_{\text {rep}}) \cdot (1 - \epsilon _{\textrm{S}_f})) \cdot (1 - p_{\text {rep}}) \cdot \epsilon _{\textrm{S}_p} \cdot (1 - \epsilon _{\textrm{S}_f}) + \\&p_{R_{p}S_{f}} \cdot (1 - \epsilon _{\textrm{S}_p}) \cdot (p_{\text {rep}} + (1 - p_{\text {rep}}) \cdot \epsilon _{\textrm{R}_f}) \cdot (1 - p_{\text {rep}}) \cdot \epsilon _{\textrm{S}_p} \cdot \epsilon _{\textrm{R}_f} +\\&p_{S_{p}R_{f}} \cdot \epsilon _{\textrm{R}_p} \cdot (p_{\text {rep}} + (1 - p_{\text {rep}}) \cdot (1 - \epsilon _{\textrm{S}_f})) \cdot (1 - p_{\text {rep}}) \cdot (1 - \epsilon _{\textrm{R}_p}) \cdot (1 - \epsilon _{\textrm{S}_f}) +\\&p_{S_{p}S_{f}} \cdot \epsilon _{\textrm{R}_p} \cdot (p_{\text {rep}} + (1 - p_{\text {rep}}) \cdot \epsilon _{\textrm{R}_f}) \cdot (1 - p_{\text {rep}}) \cdot (1 - \epsilon _{\textrm{R}_p}) \cdot \epsilon _{\textrm{R}_f} \\ \Pr (R_p,R_f,S_p,S_f) = \ &p_{R_{p}R_{f}} \cdot (1 - \epsilon _{\textrm{S}_p}) \cdot (p_{\text {rep}} + (1 - p_{\text {rep}}) \cdot (1 - \epsilon _{\textrm{S}_f})) \cdot \epsilon _{\textrm{S}_p} \cdot (p_{\text {rep}} + (1 - p_{\text {rep}}) \cdot \epsilon _{\textrm{S}_f}) + \\&p_{R_{p}S_{f}} \cdot (1 - \epsilon _{\textrm{S}_p}) \cdot (p_{\text {rep}} + (1 - p_{\text {rep}}) \cdot \epsilon _{\textrm{R}_f}) \cdot \epsilon _{\textrm{S}_p} \cdot (p_{\text {rep}} + (1 - p_{\text {rep}}) \cdot (1 - \epsilon _{\textrm{R}_f})) + \\&p_{S_{p}R_{f}} \cdot \epsilon _{\textrm{R}_p} \cdot (p_{\text {rep}} + (1 - p_{\text {rep}}) \cdot (1 - \epsilon _{\textrm{S}_f})) \cdot (1 - \epsilon _{\textrm{R}_p}) \cdot (p_{\text {rep}} + (1 - p_{\text {rep}}) \cdot \epsilon _{\textrm{S}_f}) + \\&p_{S_{p}S_{f}} \cdot \epsilon _{\textrm{R}_p} \cdot (p_{\text {rep}} + (1 - p_{\text {rep}}) \cdot \epsilon _{\textrm{R}_f}) \cdot (1 - \epsilon _{\textrm{R}_p}) \cdot (p_{\text {rep}} + (1 - p_{\text {rep}}) \cdot (1 - \epsilon _{\textrm{R}_f})) \\ \Pr (R_p,R_f,R_p,S_f) = \ &\Pr (R_p,S_f,R_p,R_f) \\ \Pr (R_p,S_f,R_p,S_f) = \ &p_{R_{p}R_{f}} \cdot (1 - \epsilon _{\textrm{S}_p}) \cdot (1 - p_{\text {rep}}) \cdot \epsilon _{\textrm{S}_f} \cdot (1 - p_{\text {rep}}) \cdot (1 - \epsilon _{\textrm{S}_p}) \cdot \epsilon _{\textrm{S}_f} + \\&p_{R_{p}S_{f}} \cdot (1 - \epsilon _{\textrm{S}_p}) \cdot (1 - p_{\text {rep}}) \cdot (1 - \epsilon _{\textrm{R}_f}) \cdot (1 - p_{\text {rep}}) \cdot (1 - \epsilon _{\textrm{S}_p}) \cdot (1 - \epsilon _{\textrm{R}_f}) + \\&p_{S_{p}R_{f}} \cdot \epsilon _{\textrm{R}_p} \cdot (1 - p_{\text {rep}}) \cdot \epsilon _{\textrm{S}_f} \cdot (1 - p_{\text {rep}}) \cdot \epsilon _{\textrm{R}_p} \cdot \epsilon _{\textrm{S}_f} + \\&p_{S_{p}S_{f}} \cdot \epsilon _{\textrm{R}_p} \cdot (1 - p_{\text {rep}}) \cdot (1 - \epsilon _{\textrm{R}_f}) \cdot (1 - p_{\text {rep}}) \cdot \epsilon _{\textrm{R}_p} \cdot (1 - \epsilon _{\textrm{R}_f}) \\ \Pr (R_p,S_f,S_p,R_f) = \ &p_{R_{p}R_{f}} \cdot (1 - \epsilon _{\textrm{S}_p}) \cdot (1 - p_{\text {rep}}) \cdot \epsilon _{\textrm{S}_f} \cdot (1 - p_{\text {rep}}) \cdot \epsilon _{\textrm{S}_p} \cdot (1 - \epsilon _{\textrm{S}_f}) + \\&p_{R_{p}S_{f}} \cdot (1 - \epsilon _{\textrm{S}_p}) \cdot (1 - p_{\text {rep}}) \cdot (1 - \epsilon _{\textrm{R}_f}) \cdot (1 - p_{\text {rep}}) \cdot \epsilon _{\textrm{S}_p} \cdot \epsilon _{\textrm{R}_f} + \\&p_{S_{p}R_{f}} \cdot \epsilon _{\textrm{R}_p} \cdot (1 - p_{\text {rep}}) \cdot \epsilon _{\textrm{S}_f} \cdot (1 - p_{\text {rep}}) \cdot (1 - \epsilon _{\textrm{R}_p}) \cdot (1 - \epsilon _{\textrm{S}_f}) +\\&p_{S_{p}S_{f}} \cdot \epsilon _{\textrm{R}_p} \cdot (1 - p_{\text {rep}}) \cdot (1 - \epsilon _{\textrm{R}_f}) \cdot (1 - p_{\text {rep}}) \cdot (1 - \epsilon _{\textrm{R}_p}) \cdot \epsilon _{\textrm{R}_f} \\ \\ \Pr (R_p,S_f,S_p,S_f) = \ &p_{R_{p}R_{f}} \cdot (1 - \epsilon _{\textrm{S}_p}) \cdot (1 - p_{\text {rep}}) \cdot \epsilon _{\textrm{S}_f} \cdot \epsilon _{\textrm{S}_p} \cdot (p_{\text {rep}} + (1 - p_{\text {rep}}) \cdot \epsilon _{\textrm{S}_f}) + \\&p_{R_{p}S_{f}} \cdot (1 - \epsilon _{\textrm{S}_p}) \cdot (1 - p_{\text {rep}}) \cdot (1 - \epsilon _{\textrm{R}_f}) \cdot \epsilon _{\textrm{S}_p} \cdot (p_{\text {rep}} + (1 - p_{\text {rep}}) \cdot (1 - \epsilon _{\textrm{R}_f})) + \\&p_{S_{p}R_{f}} \cdot \epsilon _{\textrm{R}_p} \cdot (1 - p_{\text {rep}}) \cdot \epsilon _{\textrm{S}_f} \cdot (1 - \epsilon _{\textrm{R}_p}) \cdot (p_{\text {rep}} + (1 - p_{\text {rep}}) \cdot \epsilon _{\textrm{S}_f}) + \\&p_{S_{p}S_{f}} \cdot \epsilon _{\textrm{R}_p} \cdot (1 - p_{\text {rep}}) \cdot (1 - \epsilon _{\textrm{R}_f}) \cdot (1 - \epsilon _{\textrm{R}_p}) \cdot (p_{\text {rep}} + (1 - p_{\text {rep}}) \cdot (1 - \epsilon _{\textrm{R}_f})) \\ \Pr (S_p,R_f,R_p,R_f) = \ &\Pr (R_p,R_f,S_p,R_f) \\ \Pr (S_p,R_f,R_p,S_f) = \ &\Pr (R_p,S_f,S_p,R_f) \\ \Pr (S_p,R_f,S_p,R_f) = \ &p_{R_{p}R_{f}} \cdot \epsilon _{\textrm{S}_p} \cdot (1 - p_{\text {rep}}) \cdot (1 - \epsilon _{\textrm{S}_f}) \cdot \epsilon _{\textrm{S}_p} \cdot (1 - p_{\text {rep}}) \cdot (1 - \epsilon _{\textrm{S}_f}) + \\&p_{R_{p}S_{f}} \cdot \epsilon _{\textrm{S}_p} \cdot (1 - p_{\text {rep}}) \cdot \epsilon _{\textrm{R}_f} \cdot \epsilon _{\textrm{S}_p} \cdot (1 - p_{\text {rep}}) \cdot \epsilon _{\textrm{R}_f} +\\&p_{S_{p}R_{f}} \cdot (1 - \epsilon _{\textrm{R}_p}) \cdot (1 - p_{\text {rep}}) \cdot (1 - \epsilon _{\textrm{S}_f}) \cdot (1 - \epsilon _{\textrm{R}_p}) \cdot (1 - p_{\text {rep}}) \cdot (1 - \epsilon _{\textrm{S}_f}) + \\&p_{S_{p}S_{f}} \cdot (1 - \epsilon _{\textrm{R}_p}) \cdot (1 - p_{\text {rep}}) \cdot \epsilon _{\textrm{R}_f} \cdot (1 - \epsilon _{\textrm{R}_p}) \cdot (1 - p_{\text {rep}}) \cdot \epsilon _{\textrm{R}_f} \\ \\ \end{aligned}$$

$$\begin{aligned} \Pr (S_p,R_f,S_p,S_f) = \ &p_{R_{p}R_{f}} \cdot \epsilon _{\textrm{S}_p} \cdot (1 - p_{\text {rep}}) \cdot (1 - \epsilon _{\textrm{S}_f}) \cdot \epsilon _{\textrm{S}_p} \cdot (p_{\text {rep}} + (1 - p_{\text {rep}}) \cdot \epsilon _{\textrm{S}_f}) + \\&p_{R_{p}S_{f}} \cdot \epsilon _{\textrm{S}_p} \cdot (1 - p_{\text {rep}}) \cdot \epsilon _{\textrm{R}_f} \cdot \epsilon _{\textrm{S}_p} \cdot (p_{\text {rep}} + (1 - p_{\text {rep}}) \cdot (1 - \epsilon _{\textrm{R}_f})) + \\&p_{S_{p}R_{f}} \cdot (1 - \epsilon _{\textrm{R}_p}) \cdot (1 - p_{\text {rep}}) \cdot (1 - \epsilon _{\textrm{S}_f}) \cdot (1 - \epsilon _{\textrm{R}_p}) \cdot (p_{\text {rep}} + (1 - p_{\text {rep}}) \cdot \epsilon _{\textrm{S}_f}) + \\&p_{S_{p}S_{f}} \cdot (1 - \epsilon _{\textrm{R}_p}) \cdot (1 - p_{\text {rep}}) \cdot \epsilon _{\textrm{R}_f} \cdot (1 - \epsilon _{\textrm{R}_p}) \cdot (p_{\text {rep}} + (1 - p_{\text {rep}}) \cdot (1 - \epsilon _{\textrm{R}_f})) \\ \\ \Pr (S_p,S_f,R_p,S_f) = \ &\Pr (R_p,S_f,S_p,S_f) \\ \\ \Pr (S_p,S_f,R_p,S_f) = \ &\Pr (R_p,S_f,S_p,S_f) \\ \\ \Pr (S_p,S_f,S_p,R_f) = \ &\Pr (S_p,R_f,S_p,S_f) \\ \\ \Pr (S_p,S_f,S_p,S_f) = \ &p_{R_{p}R_{f}} \cdot \epsilon _{\textrm{S}_p} \cdot (p_{\text {rep}} + (1 - p_{\text {rep}}) \cdot \epsilon _{\textrm{S}_f}) \cdot \epsilon _{\textrm{S}_p} \cdot (p_{\text {rep}} + (1 - p_{\text {rep}}) \cdot \epsilon _{\textrm{S}_f}) + \\&p_{R_{p}S_{f}} \cdot \epsilon _{\textrm{S}_p} \cdot (p_{\text {rep}} + (1 - p_{\text {rep}}) \cdot (1 - \epsilon _{\textrm{R}_f})) \cdot \epsilon _{\textrm{S}_p} \cdot (p_{\text {rep}} + (1 - p_{\text {rep}}) \cdot (1 - \epsilon _{\textrm{R}_f})) + \\&p_{S_{p}R_{f}} \cdot (1 - \epsilon _{\textrm{R}_p}) \cdot (p_{\text {rep}} + (1 - p_{\text {rep}}) \cdot \epsilon _{\textrm{S}_f}) \cdot (1 - \epsilon _{\textrm{R}_p}) \cdot (p_{\text {rep}} + (1 - p_{\text {rep}}) \cdot \epsilon _{\textrm{S}_f}) + \\&p_{S_{p}S_{f}} \cdot (1 - \epsilon _{\textrm{R}_p}) \cdot (p_{\text {rep}} + (1 - p_{\text {rep}}) \cdot (1 - \epsilon _{\textrm{R}_f})) \cdot (1 - \epsilon _{\textrm{R}_p}) \cdot (p_{\text {rep}} + (1 - p_{\text {rep}}) \cdot (1 - \epsilon _{\textrm{R}_f})) \end{aligned}$$

## Appendix B A Critical Inequality of $\mathcal {M}_{\text {RPPH}}$

In this theorem, we prove a critical inequality of $\mathcal {M}_{\text {RPPH}}$ between joint response probabilities.

### Theorem 1

For $\mathcal {M}_{\text {RPPH}}$, if $p_{S_{p}S_{f}} = 1$, then $\Pr (S_p,S_f,S_p,S_f) \ge \Pr (R_p,R_f,R_p,R_f)$.

### Proof

We will begin by assuming that $p_{S_{p}S_{f}} = 1$ and noting a few facts to support the proof. First, the terms below are constrained within the following range:

A4 $$\begin{aligned} 0 \le \epsilon _{\textrm{S}_p},\epsilon _{\textrm{S}_f},\epsilon _{\textrm{R}_p},\epsilon _{\textrm{R}_f} \le .50 \end{aligned}$$

Based on the constraints described in Inequality A4, the following inequalities between complimentary response terms must hold:

A5 $$\begin{aligned} \epsilon _{\textrm{R}_p} \le (1 - \epsilon _{\textrm{R}_p}) \end{aligned}$$

A6 $$\begin{aligned} \epsilon _{\textrm{R}_f} \le (1 - \epsilon _{\textrm{R}_f}) \end{aligned}$$

Under the assumption that $p_{S_{p}S_{f}} = 1$, the joint probabilities specified in Theorem 1 simplify to the following:

A7 $$\begin{aligned} \Pr (S_p,S_f,S_p,S_f) = p_{S_{p}S_{f}}&\cdot (1 - \epsilon _{\textrm{R}_p}) \cdot (p_{\text {rep}} + (1 - p_{\text {rep}})\nonumber \\ &\cdot (1 - \epsilon _{\textrm{R}_f})) \cdot (1 - \epsilon _{\textrm{R}_p}) \nonumber \\ &\cdot (p_{\text {rep}} + (1 - p_{\text {rep}}) \cdot (1 - \epsilon _{\textrm{R}_f})) \end{aligned}$$

A8 $$\begin{aligned} \Pr (R_p,R_f,R_p,R_f) = p_{S_{p}S_{f}}&\cdot \epsilon _{\textrm{R}_p} \cdot (p_{\text {rep}} + (1 - p_{\text {rep}}) \cdot \epsilon _{\textrm{R}_f}) \nonumber \\ &\cdot \epsilon _{\textrm{R}_p} \cdot (p_{\text {rep}} + (1 - p_{\text {rep}}) \cdot \epsilon _{\textrm{R}_f}) . \end{aligned}$$

Importantly, the structure of Eq. 47 is identical to that of Eq. 48, except the response terms in one equation are the complement of the response terms in the other equation. For example, Eq. 47 becomes identical to Eq. 48 if the response terms $(1 - \epsilon _{\textrm{R}_p})$ and $(1 - \epsilon _{\textrm{R}_f})$ are replaced with $\epsilon _{\textrm{R}_p}$ and $\epsilon _{\textrm{R}_f}$, respectively. For this reason, we can prove the inequality in Theorem 1 by comparing the relationship between complementary response terms using the inequalities defined in 46 and 45. To complete the proof, we note that response terms in Eqs. 47 and 48 are combined through multiplication, all terms are probabilities and therefore non-negative, which means the joint probability with the larger response terms will be greater than or equal to the other joint probability. Because the definitions of $\Pr (S_p,S_f,S_p,S_f)$ and $\Pr (R_p,R_f,R_p,R_f)$ differ only in response terms, and by Inequalities 45 and 46, the response terms in $\Pr (S_p,S_f,S_p,S_f)$ are greater than or equal to those in $\Pr (R_p,R_f,R_p,R_f)$, it follows that

$$\begin{aligned} \Pr (S_p,S_f,S_p,S_f) \ge \Pr (R_p,R_f,R_p,R_f), \end{aligned}$$

which proves the inequality stated in the theorem. $\square$

## Footnotes

1 TEM does not imply responses between choice sets are independent in general (Birnbaum, 2013).

## Code Availability

Code is available at https://github.com/itsdfish/dynamic_inconsistency_model_comparison
